# Prolonged treatment with the proteasome inhibitor MG-132 induces apoptosis in PC12 rat pheochromocytoma cells

**DOI:** 10.1038/s41598-022-09763-z

**Published:** 2022-04-06

**Authors:** Oktávia Tarjányi, Julian Haerer, Mónika Vecsernyés, Gergely Berta, Alexandra Stayer-Harci, Bálint Balogh, Kornélia Farkas, Ferenc Boldizsár, József Szeberényi, György Sétáló

**Affiliations:** 1 Department of Medical Biology and Central Electron Microscope Laboratory, University of Pécs, Medical School, Szigeti út 12., Pecs, 7624 Hungary; 2 Signal Transduction Research Group, János Szentágothai Research Centre, Ifjúság útja 20., Pecs, 7624 Hungary; 3 Institute of Bioanalysis, University of Pécs, Medical School, Szigeti út 12., Pecs, 7624 Hungary; 4 Department of Immunology and Biotechnology, University of Pécs, Medical School, Szigeti út 12., Pecs, 7624 Hungary

**Keywords:** Molecular medicine, Apoptosis, Growth factor signalling, Stress signalling, Proteasome

## Abstract

Rat pheochromocytoma (PC12) cells were treated with the proteasome inhibitor MG-132 and morphological changes were recorded. Initially, neuronal differentiation was induced but after 24 h signs of morphological deterioration became apparent. We performed nuclear staining, flow cytometry and WST-1 assay then analyzed signal transduction pathways involving Akt, p38 MAPK (Mitogen-Activated Protein Kinase), JNK (c-Jun N-terminal Kinase), c-Jun and caspase-3. Stress signaling via p38, JNK and c-Jun was active even after 24 h of MG-132 treatment, while the survival-mediating Akt phosphorylation declined and the executor of apoptosis (caspase-3) was activated by that time and apoptosis was also observable. We examined subcellular localization of stress signaling components, applied kinase inhibitors and dominant negative H-Ras mutant-expressing PC12 cells in order to decipher connections of stress-mediating pathways. Our results are suggestive of that treatment with the proteasome inhibitor MG-132 has a biphasic nature in PC12 cells. Initially, it induces neuronal differentiation but prolonged treatments lead to apoptosis.

## Introduction

Rat pheochromocytoma (PC12) cells are a popular model to study neuronal differentiation, survival and apoptosis. They are round-shaped and proliferate readily under normal culturing conditions. Upon exposure to nerve growth factor (NGF) they stop to divide, grow projections (neurites) and differentiate into a sympathetic neuron-like phenotype. Besides NGF other agents can also induce neuritogenesis in these cells, for example the proteasome inhibitor MG-132 within approximately 24 h^1^. However, longer treatments with this compound do not cause further differentiation, on the contrary, the morphology of the cells quickly deteriorates. The signaling mechanisms behind the morphological alterations are not yet fully understood.

The ubiquitin–proteasome system (UPS) plays a critical role in the breakdown of unnecessary, misfolded or damaged proteins^2^ and it regulates the activation or inactivation of various signaling molecules involved in cell cycle control, inflammation, apoptosis or differentiation^3–5^. Around 80% of intracellular proteins are degraded by this mechanism in a well-regulated manner. Poly-ubiquitinated proteins are recognized and broken down by a cylindrical multicatalytic proteinase complex called the 26S proteasome. It consists of a 20S catalytic core and two 19S regulatory caps. The 20S catalytic core is organized into two α and two β rings. Three subunits of the β-rings (β1, β2, β5) are responsible for the proteolytic activities of the proteasome^6^. The dysfunction of the UPS is involved in numerous pathological conditions such as inflammation^7^, tumors^8^ or neurological diseases^9^.

Several inhibitors have been developed to block the function of the proteasome either in a reversible- or irreversible fashion^10^. Some of these compounds have already been approved for the treatment of hematological malignancies and their field of indication is likely to broaden further. At the same time, in case of some proteasome inhibitors, peripheral neuropathy was reported as a therapeutic side effect^11^, although the exact mechanism of it is still not understood.

In our previous work^1^ we treated PC12 cells with 2.5 μM MG-132 for various time periods and examined the morphological and intracellular signaling events induced. Initially, we could see neuronal differentiation (neuritogenesis) but after 24 h of MG-132 treatment substantial morphological changes occurred to the cells fairly rapidly: the neurites shrank, the cells were getting again rounder, their adherence to the culture dish became weaker and an increasing number of cells tended to float in groups in the culturing medium. In the present study we focused our attention onto the process of possible cellular stress and apoptosis in PC12 cells as a result of MG-132 treatments longer than 24 h (prolonged treatment).

Here we report that prolonged exposure of PC12 cells to the proteasome inhibitor MG-132 causes cell death by apoptosis. At the molecular level—besides the previously reported differentiative signaling events^1^—we identified a slow but sustained phosphorylation of the signaling components Akt, p38 MAPK (Mitogen-Activated Protein Kinase), JNK (c-Jun N-terminal Kinase) and c-Jun. After 24 h of treatment the survival signals declined, while stress signaling via p38 and JNK was still highly active. Additionally, caspase-3 cleavage is also detectable upon 24 h of MG-132 treatment of the cells. The observed shift towards stress signaling and the consequential caspase-3 activation is likely to contribute to the programmed death of PC12 cells induced by prolonged proteasome inhibitor treatment.

## Materials and methods

### Reagents

All fine chemicals were purchased from Sigma (Saint Louis, MO, USA) unless indicated otherwise. MG-132 (Merck, Kenilworth, NJ, USA Cat. # 474790) used at a final concentration of 2.5 µM is a potent and selective, cell permeable, peptidyl-aldehyde type proteasome inhibitor that blocks the enzymatic activity of the proteasome reversibly. Hoechst 33342 (Calbiochem, La Jolla, CA, USA) is a fluorescent DNA-dye used to visualize the nuclei of cells. AnnexinV-FITC (BD Biosciences, San Jose, CA, USA Cat. # 556419) and propidium-iodide (PI) are used to identify apoptotic cells. WST-1 (Roche, Mannheim, Germany, Cat. # 11 644 807 001) is a tetrazolium salt used for the determination of the ratio of living cells in samples based on the activity of mitochondrial dehydrogenases which convert WST-1 to formazan. LY294002 (Cell Signaling Technology, Danvers, MA, USA, Cat. # 9901) used at a concentration of 20 μM is a highly selective inhibitor of phosphatidylinositol 3 kinase (PI3K). It blocks the PI3K-dependent phosphorylation and kinase activity of Akt. SB203580 (Cat. # S8307) used at 10 μM concentration is a selective p38 MAPK inhibitor that blocks the catalytic activity of p38 by interfering with the ATP binding pocket of the enzyme. SP600125 (Cat. # S5567) used at 50 μM concentration is a potent and selective ATP-competitive inhibitor of JNK-1, -2 and -3. The Western blot lysis buffer contained 50 mM Tris base (pH 7.4), 150 mM NaCl, 10% glycerol, 1 mM EGTA, 1 mM Na-orthovanadate, 5 mM ZnCl_2_, 100 mM NaF, 10 mg/ml aprotinin, 1 mg/ml leupeptin, 1 mM PMSF and 1% Triton X-100. The Western blot sample buffer contained 7 ml 4 × Tris–HCl/SDS (pH: 6.8), 3.8 g (~ 3 ml) glycerol, 1 g SDS, 0.93 g dithiothreitol (DTT), 1.2 mg bromophenol blue dissolved in 10 ml distilled water (dH_2_O). TBS-Tween for Western blotting contained: 10 mM Tris-base, 150 mM NaCl, 0.2% Tween-20, pH 8.0. The Western blot stripping buffer contained 0.2 M glycine–HCl (pH 2.5), 0.05% Tween 20 dissolved in 1 L dH_2_O. The 4x-concentrated Tris–HCl/SDS solution (pH: 6.8) contained 6.05 g Tris base, 0.4 g SDS dissolved in 100 ml dH_2_O. For immunofluorescence the TBS buffer contained 50 ml 10 × Tris HCl, 15 ml 5 M NaCl dissolved in 435 ml dH_2_O. The TBS-T buffer contained 0.1% Triton X-100 dissolved in TBS. The PBS buffer (pH 7.4) contained 1.37 mM NaCl, 0.27 mM KCl, 0.43 mM Na_2_HPO_4_ 7H_2_O, 0.14 mM KH_2_PO_4_. For fixation, 4% paraformaldehyde was dissolved in PBS (pH 7.4). AnnexinV-FITC and PI staining was performed in Annexin-binding buffer containing 0.01 M Hepes (pH 7.4), 0.14 M NaCl and 2.5 mM CaCl_2_. The proteasome activity was measured by the 20S Proteasome Activity Assay Kit (Merck, Darmstadt, Germany, Cat. # APT280). Lysis buffer for the Proteasome Activity Assay Kit contained 50 mM HEPES (pH 7.5), 5 mM EDTA, 150 mM NaCl and 1% Triton X-100.

### Cells

Wild type PC12 and M-M17-26 (dominant negative H-Ras protein-expressing)^12^ rat pheochromocytoma cells were cultured in DMEM containing 5% fetal calf serum and 10% heat-inactivated horse serum (GIBCO, Paisley, Scotland). Both cell lines were kindly provided by G.M. Cooper (Department of Biology, Boston University, MA).

### Proteasome inhibitor and kinase inhibitor treatments, measurement of proteasome activity

10^6^ cells were seeded onto Petri dishes or plastic cover slips. Treatments were started when the cells adhered to the surface and presented healthy phenotype 1 day after plating. First, cells were starved in 0.5% horse serum-containing medium for 24 h to reduce mitogenic signals. Treatments of various lengths (5-, 15-, 30 min, 1-, 3-, 6-, 24-, 28-, 30- and 48 h) with the proteasome inhibitor (MG-132) at 2.5 µM concentration were started so that all cultures could be terminated simultaneously. To check the effectivity and kinetics of MG-132 treatments, we measured the proteasome activity in PC12 cells (Supplementary Fig. 1). 20 µl from the cell lysates of 5 × 10^6^ cells were used from each sample. The assay was carried out according to the manufacturer’s instructions. Lactacystin was used as positive control, representing maximal proteasome inhibition. The fluorescent end product was measured by a BMG Labtech CLARIOstar Plus microplate reader (BMG Labtech, Offenburg, Germany) at 380/460 nm. Kinase inhibitors (LY294002, SB203580, SP600125) were added to the cultures 1 h prior to the proteasome inhibitor so that both compounds were present during the 3-h-long MG-132 treatment.

### Antibodies

The following primary antibodies (all from Cell Signaling Technology, Danvers, MA, USA) were used: Phospho-Akt (Ser473) (D9E) XP rabbit mAb (Cat. # 4060): 1:1000 (Western blot), Akt (Cat. # 9272) at 1:1000 (Western blot), Phospho-p38 MAPK (Thr180/Tyr182) (Cat. # 9211) at 1:1000 (Western blot) or 1:200 (immunofluorescence), p38 MAPK (Cat. # 9212) at 1:1000 (Western blot), Phospho-SAPK/JNK (Thr183/Tyr185) (Cat. # 9251) at 1:1000 (Western blot) or 1:200 (immunofluorescence), SAPK/JNK (Cat. # 9252) at 1:1000 (Western blot), Phospho-c-Jun (Ser73) (Cat. # 9164) at 1:1000 (Western blot) or 1:200 (immunofluorescence), c-Jun (60A8) Rabbit mAb (Cat. # 9165) at 1:1000 (Western blot), Cleaved Caspase-3 (Asp175) (Cat. # 9661) at 1:1000 (Western blot), GAPDH (D16H11) XP Rabbit mAb (Cat. # 5174) at 1:5000 (Western blot). HRP-conjugated goat anti-rabbit IgG (Pierce Biotechnology, Rockford, IL, USA, Cat# 31460) was used at 1:10 000 for Western blots and Cy3-conjugated goat anti-rabbit IgG (Jackson ImmunoResearch Laboratories, West Grove, PA, USA Cat# 111-165-144) at 1:600 for immunofluorescence as secondary antibodies.

### Phase contrast microscopy

Phenotypic changes of PC12 cells, cultured under the indicated conditions, were analyzed using an Olympus FluoView-1000 laser scanning confocal fluorescence microscope in combined phase contrast mode.

### Nuclear staining

After fixation in 4% paraformaldehyde (see Reagents above) and permeabilization (in TBS-T) cells were incubated with Hoechst 33342 for 10 min at room temperature. Nuclear morphology was documented with an Olympus FluoView-1000 laser scanning confocal fluorescence microscope. Chromatin condensation and nuclear fragmentation were considered as signs of apoptosis. 100 cells were counted per sample and the percentage of apoptotic cells was calculated.

### Flow cytometry

Adherent PC12 cells were collected using trypsin. After 2 washes with PBS AnnexinV-FITC and PI staining was performed according to the manufacturer's instructions in order to distinguish apoptotic and living cells. Briefly, 10^5^ cells were incubated with 5 μl AnnexinV-FITC for 15 min in 100 μl Annexin-binding buffer. Then the samples were diluted with 400 μl Annexin-binding buffer and 1 μl PI was added to the samples immediately before the flow cytometric analysis. Samples were analyzed with a FACS Calibur (BD Biosciences, San Jose, CA, USA) flow cytometer using the Cell Quest (BD Biosciences, San Jose, CA, USA) software. AnnexinV^−^PI^−^, AnnexinV^+^PI^−^ and AnnexinV^+^PI^+^ cell populations were distinguished based on their FL1/FL2 fluorescence and annotated as living-, early apoptotic- and late apoptotic cells, respectively.

### WST-1 cell viability assay

The WST-1 colorimetric assay for the quantification of cell viability was used according to the manufacturer's instructions. Briefly, 2 × 10^4^ cells after their MG-132 treatment were incubated further in the presence of WST-1 for 4 h at 37 °C. Living cells with their mitochondrial enzymes convert the tetrazolium salt into formazan, a colorful product. The amount of produced formazan correlates with the number of metabolically active/living cells in the cultures. The O.D. of samples were measured at 450 nm with a BMG Labtech Fluostar Optima microplate reader (BMG Labtech, Offenburg, Germany).

### Western blot

For Western blots the cells were collected by scraping into the culturing media. After centrifugation for 5 min at 1000 rpm at 4 °C the medium was discarded and the pellet was lysed in 100 µl ice cold lysis buffer for 30 min. Samples were centrifuged at 13,500 rpm for 30 min at 4 °C to remove the insoluble cell fraction. The protein concentration of the supernatants was determined (Lowry’s method, Detergent Compatible Protein Assay Kit, Bio-Rad, Hercules, CA, USA) and SDS-containing sample buffer was added to equal amounts of proteins then boiled for 5 min for denaturation. Samples were separated in SDS-containing 10% polyacrylamide gels, then transferred onto PVDF membranes with a BioRad Trans-Blot^®^ Turbo™ Transfer System. Blotted membranes were blocked with 3% BSA or 5% milk (in case of Akt, cleaved caspase-3 and GAPDH) in TBS-Tween and incubated with the primary antibodies overnight at 4 °C in blocking solution, washed 3 times and then incubated with horseradish peroxidase-conjugated secondary antibodies for 1 h at room temperature in blocking solution again. Immune complexes were visualized using ECL reagent (Millipore Immobilon Western Chemiluminescent HRP substrate). Chemiluminescent signals were detected using the G:Box gel documentation system (Syngene, Cambridge, UK). To remove the bound antibodies, membranes were washed with hot stripping buffer for 2 × 20 min and were re-probed using the primary antibodies against the unphosphorylated forms of the signaling molecules or GAPDH to check for equal loading of the samples.

### Immunofluorescence and confocal microscopy

Cells were cultured on plastic Thermanox cover slips (Nalgene Nunc International, Rochester, NY, USA). Treatments were stopped by rinsing in 37 °C PBS followed by fixation at room temperature for 1 h in 4% paraformaldehyde dissolved in PBS. Excess fixative was washed out 3 × with PBS and 3 × with TBS followed by 1 h permeabilization in TBS-T. The blocking of nonspecific binding sites was carried out by incubating the samples in 3% BSA dissolved in TBS-T. After incubation with the primary antibodies (overnight, at 4 °C) samples were washed 5 × in TBS-T. Then the samples were incubated with the Cy3-conjugated secondary antibodies in dark for 1 h, at 4 °C. Five washes in TBS-T removed the unbound antibodies. Nuclei were counterstained with Hoechst 33342. Finally, preparations were covered with Vectashield (Vector Laboratories, Burlingame, CA). Fluorescent signals were detected by laser scanning confocal microscopy using the Olympus FluoView-1000 system with a 40 × phase objective*.*

### Statistical analysis

To detect differences between the effects of treatments one-way ANOVA test was applied, to eliminate the type I error Bonferroni’s post hoc tests were used in all cases. Data are represented as mean and standard deviation (SD). A p-value less than 0.05 was considered as significant. Statistical analyses were performed with IBM SPSS Statistics v 24.0 software package (IBM’s Corporate, New York, USA).

## Results

### Apoptosis of PC12 cells treated with MG-132 for 24 h or longer

**Figure 1:**
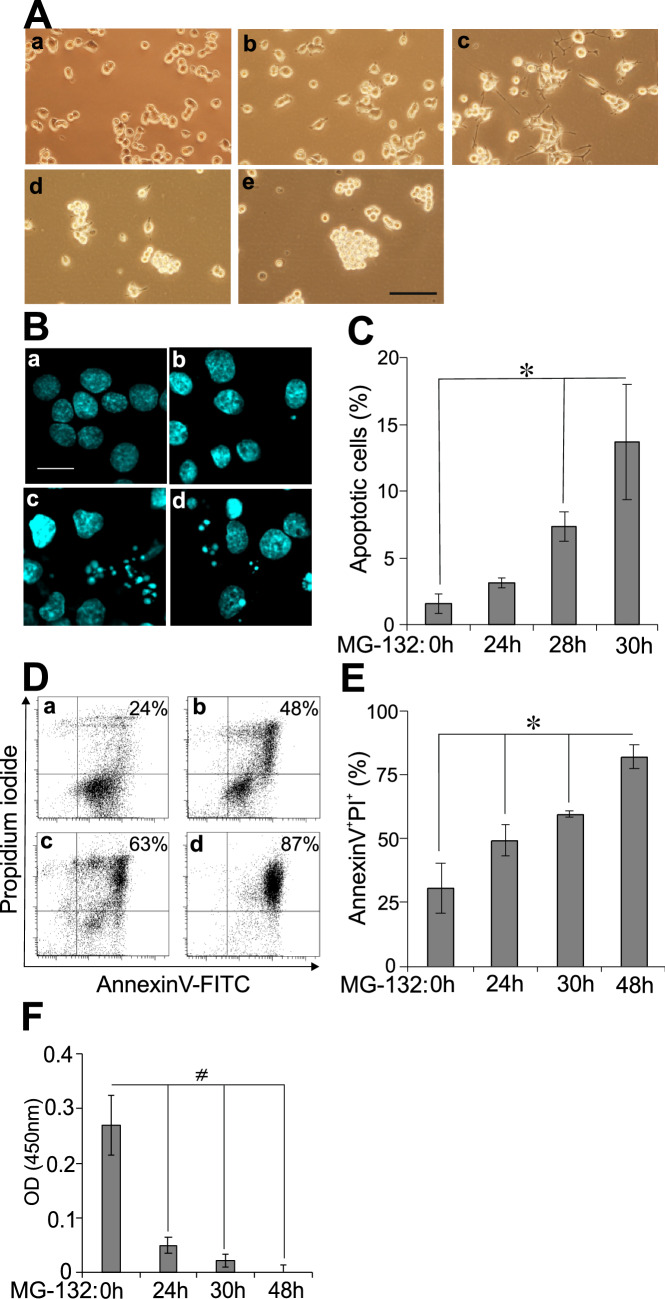
Prolonged MG-132 treatment induces apoptosis in PC12 cells. (**A**) Representative phase contrast images show PC12 cells after 0 (**Aa**), 6 (**Ab**), 24 (**Ac**), 28 (**Ad**) and 30 (**Ae**) hours of MG-132 (2.5 µM) treatment. The scalebar (200 μm) in panel (e) applies to all phase contrast images (a–e) (**B**) Representative laser scanning confocal fluorescence microscopic images of PC12 cells’ nuclei in response to 0 (**Ba**), 24 (**Bb**), 28 (**Bc**), and 30 (**Bd**) hours of MG-132 (2.5 µM) treatment. Nuclei were stained with the fluorescent DNA-binding dye, Hoechst 33342. Chromatin condensation and fragmentation were considered as apoptotic morphological changes. The scalebar in panel (a) (20 μm) applies to all images (a–d) with fluorescent nuclei. Note, at 48 h most of the cells floated off from the slides making it impossible technically to take informative images. (**C**) Quantitative analysis of apoptotic cells detected with Hoechst 33342 staining (see micrographs under **B**) at different time points of the experiment. The percentage of apoptotic cells was determined as the ratio of cells with abnormal- versus normal chromatin structure. Column diagram shows the mean ± SD values calculated from the data of three independent experiments. Statistically significant differences are indicated (*P ≤ 0.05). (**D**) Flow cytometric analysis of MG-132 treatment-induced apoptosis in PC12 cells. Cells were stained with Annexin V-FITC/PI after 0 (**Da**), 24 (**Db**), 30 (**Dc**) and 48 (**Dd**) hours of MG-132 (2.5 µM) treatment. Annexin V^+^ Propidium-iodide (PI)^+^ double positive cells (upper right quadrant in the representative dot-plots) were considered late apoptotic (non-viable). Percentages of dead cells are indicated in the upper right corner of each sample. (**E**) Bar diagram showing the mean ratio ± SD values calculated from the flow cytometric analysis (see **D**) of three independent experiments. Statistically significant differences are indicated (*P ≤ 0.05). (**F**) Cell viability measured using WST-1 assay. At the end of the experiment the amount of formazan dye in the samples correlates with the number of living cells in the cultures. Bars show formazan optical density measured at 450 nm after various incubation periods with MG-132 (2.5 µM). Statistically significant differences are indicated (^#^P ≤ 0.05).

Previously we treated PC12 cells with the proteasome inhibitor MG-132 for various lengths of time up to one day and experienced neuronal differentiation^1^. After 24 h, however, a decline in overall cell morphology was increasingly apparent. The observed phenomena inspired us to examine further what happens during prolonged proteasome inhibitor treatment of PC12 cells at the morphological level and regarding stress and apoptosis signaling. First, we monitored time-dependent changes of PC12 cells’ morphology upon MG-132 treatment by means of phase contrast microscopy (Fig. 1A). Untreated PC12 cells are round-shaped (Fig. 1Aa). After 6 h of MG-132 treatment small projections were detectable as a sign of beginning neuritogenesis (Fig. 1Ab). At 24 h of treatment long neurites were visible (Fig. 1Ac), while after 24 h the signs of neuronal differentiation started to diminish (neurites became shorter) and the cells began to lose their adherence to the culturing plates. After 28 h in the presence of the proteasome inhibitor (Fig. 1Ad) the cells tended to float in groups in the culturing medium, which phenomenon became even more prominent after 30 h (Fig. 1Ae). Based on the observed morphological alterations we became intrigued in the possible mechanisms in their background.

We performed Hoechst 33342 staining and analyzed nuclear morphology of the cells using laser scanning confocal fluorescence microscopy (Fig. 1B). The nuclei of PC12 cells remained mostly intact up to 24 h of MG-132 treatment (Fig. 1Bb), however, chromatin condensation and nuclear fragmentation became more and more prominent after 28 or 30 h of treatment (Fig. 1Bc and Bd, respectively) when compared to the untreated control (Fig. 1Ba). We considered chromatin condensation and nuclear fragmentation as apoptotic morphological changes. To quantify these nuclear alterations 100 cells per sample were counted and the ratio of nuclei with apoptotic morphology was calculated (Fig. 1C). The proportion of dying cells increased significantly over time (Fig. 1C). In case of apoptotic cell nuclei (Fig. 1B,C), unfortunately, we couldn’t evaluate the results after 48 h of MG-132 treatment, because almost all cells were severely affected by the treatment, hence floated off the slides during preparation of the microscopic samples. Consequently, only empty, or almost empty fields could be visualized in the microscope, this way not allowing a proper statistical analysis of the results. This is the reason why no image and no numerical ratio of apoptotic cells are indicated in Fig. 1B,C.

We complemented the above morphological analysis with flow cytometric measurements. The combined use of AnnexinV and propidium-iodide (PI) is a reliable way of determining apoptotic cell rate by means of flow cytometry. We stained PC12 cells with Annexin V-FITC/PI after 0 (Fig. 1Da), 24 (Fig. 1Db), 30 (Fig. 1Dc) and 48 (Fig. 1Dd) hours of MG-132 treatment. We considered Annexin V^+^ PI^+^ double positive cells late apoptotic (non-viable). The percentage of dead cells were determined in individual dot-plots and we collected the data of 3 independent experiments (Fig. 1E). There was a significant increase in the ratio of Annexin V^+^ PI^+^ double positive cells after 24, 30 or 48 h of MG-132 treatment (Fig. 1E). As expected, with flow cytometry we measured a higher percentage of apoptotic cells than with confocal fluorescence microscopy (see above) because here not only the adherent cells were involved in the analysis but those already floating in the media as well. Additionally, since our experiments were carried out with adherent PC12 cells, for the flow cytometric analysis trypsin had to be used to collect them, which enhances phosphatidyl-serine (PS) externalization in the membrane^13,14^ and, as a result of it, can also elevate Annexin V staining of the cells.

Finally, in order to complete the evaluation of apoptotic changes in PC12 cells upon prolonged MG-132 treatment, we performed a cell viability test using the cell proliferation indicator WST-1 (Fig. 1F). Briefly, in living cells the tetrazolium salt WST-1 is converted by mitochondrial dehydrogenases into formazan, the amount of which correlates with the number of metabolically active/living cells in the cultures. It is worth mentioning that due to protocol requirements of the WST-1 assay, upon completion of the MG-132 treatments the media were removed. Then the cells had to be incubated for an additional 4 h with the tetrazolium salt in a newly added buffer which did not contain MG-132. Nevertheless, since MG-132 had already been taken up into the cells by this time, in these cases, due to the 4-h-long additional incubation requirement with the tetrazolium salt the total duration of the MG-132 effect was practically also extended by 4 h (see Supplementary Fig. 1). This circumstance explains very likely the dramatic drop in mitochondrial activity in the sample treated for 24 h with MG-132 compared to the untreated control. We measured the optical density (OD) of formazan in samples treated with MG-132 for 0, 24, 30 and 48 h (Fig. 1F). In accordance with our earlier findings with Hoechst and Annexin V/PI staining and perhaps even more prominently the proportion of metabolically active cells decreased significantly already after 24, then at 30 and 48 h of MG-132 treatment (Fig. 1F).

Before proceeding further, we wanted to confirm the effectiveness and kinetics of our MG-132 treatment. For this reason, we performed an additional proteasome activity assay (Supplementary Fig. 1). MG-132 treatment resulted almost in 80% decrease of the proteasome activity in PC12 cells (Supplementary Fig. 1). Proteasome inhibition was evident already after 3 h of treatment and remained at this suppressed level without significant alterations even during our longest incubation with the compound (48 h) (Supplementary Fig. 1).

### Time kinetics of Akt phosphorylation, the activation of stress signaling molecules and caspase-3 cleavage induced by MG-132 treatment

**Figure 2:**
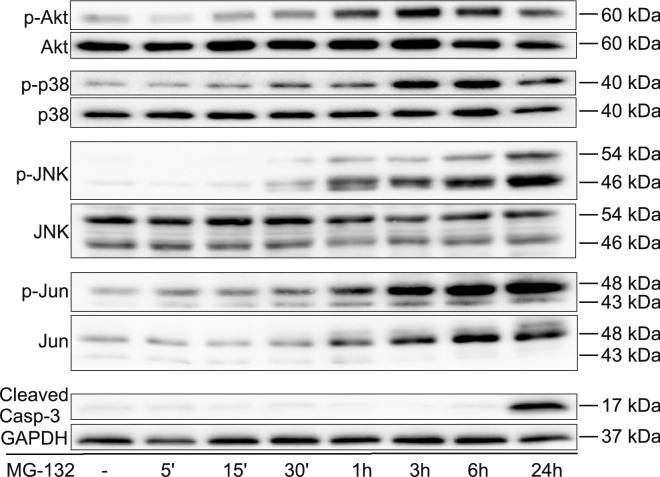
Time kinetics of MG-132-induced Akt, p38, JNK, c-Jun phosphorylation and caspase-3 cleavage. Representative Western blots show the phosphorylation changes of Akt, p38, JNK and c-Jun or the activating cleavage of caspase-3 after 0, 5, 15, 30 min or 1, 3, 6 and 24 h of MG-132 (2.5 µM) treatment. Blots (except for caspase-3) were first probed with phospho-specific antibodies (upper panels), followed by stripping and re-probing with the antibodies specific for the non-phosphorylated forms of the proteins (lower panels). The GAPDH signal was loading reference for cleaved caspase-3. All blots were cropped, original membrane images are shown in Supplementary Fig. 4. All experiments were repeated with similar results.

Having seen that after 24 h of MG-132 treatment an increasing portion of PC12 cells started to enter apoptosis, we decided to examine the signaling background of this process. Since the proteasome is involved in the regulation of numerous signaling molecules, we analyzed some of the key pathways that influence survival, mediate stress or even apoptosis of cells. First, we analyzed the phosphorylation of Akt where MG-132 induced a phosphorylation peak at 3 h of treatment. Afterwards, the declining signal followed up to 48 h (Fig. 2 and Supplementary Fig. 2).

Stress signaling pathways also play a pivotal role in regulating the apoptosis of various cell types^15^, including PC12 cells^16^. So next, we examined the phosphorylation of p38 MAPK and that of JNK and its substrate, c-Jun upon MG-132 treatment (Fig. 2). The peak of p38 phosphorylation lasted from 3 to 6 h of MG-132 treatment. Then the signal became weaker but remained still higher than in the control sample up to 48 h (Fig. 2 and Supplementary Fig. 2). The activation of JNK was apparent after the first 30 min already and it continued to increase up to 48 h of MG-132 treatment (Fig. 2 and Supplementary Fig. 2). A similar pattern was detectable with c-Jun, moreover, its unphosphorylated version showed the same increasing signal kinetics (Fig. 2).

As for caspase-3, its activating cleavage could be detected in the sample after 24 h of MG-132 treatment (Fig. 2). The cleaved caspase-3 signal became even more pronounced after 30 and 48 h (Supplementary Fig. 2).

In summary, after these extended periods of MG-132 treatment the survival-mediating Akt activation declined permanently, whereas the apoptosis-inducing stress signaling pathways were becoming more and more active, and a robust elevation of cleaved caspase-3 was also observable in PC12 cells (Supplementary Fig. 2).

### Subcellular distribution of stress signaling molecules upon MG-132 treatment

**Figure 3:**
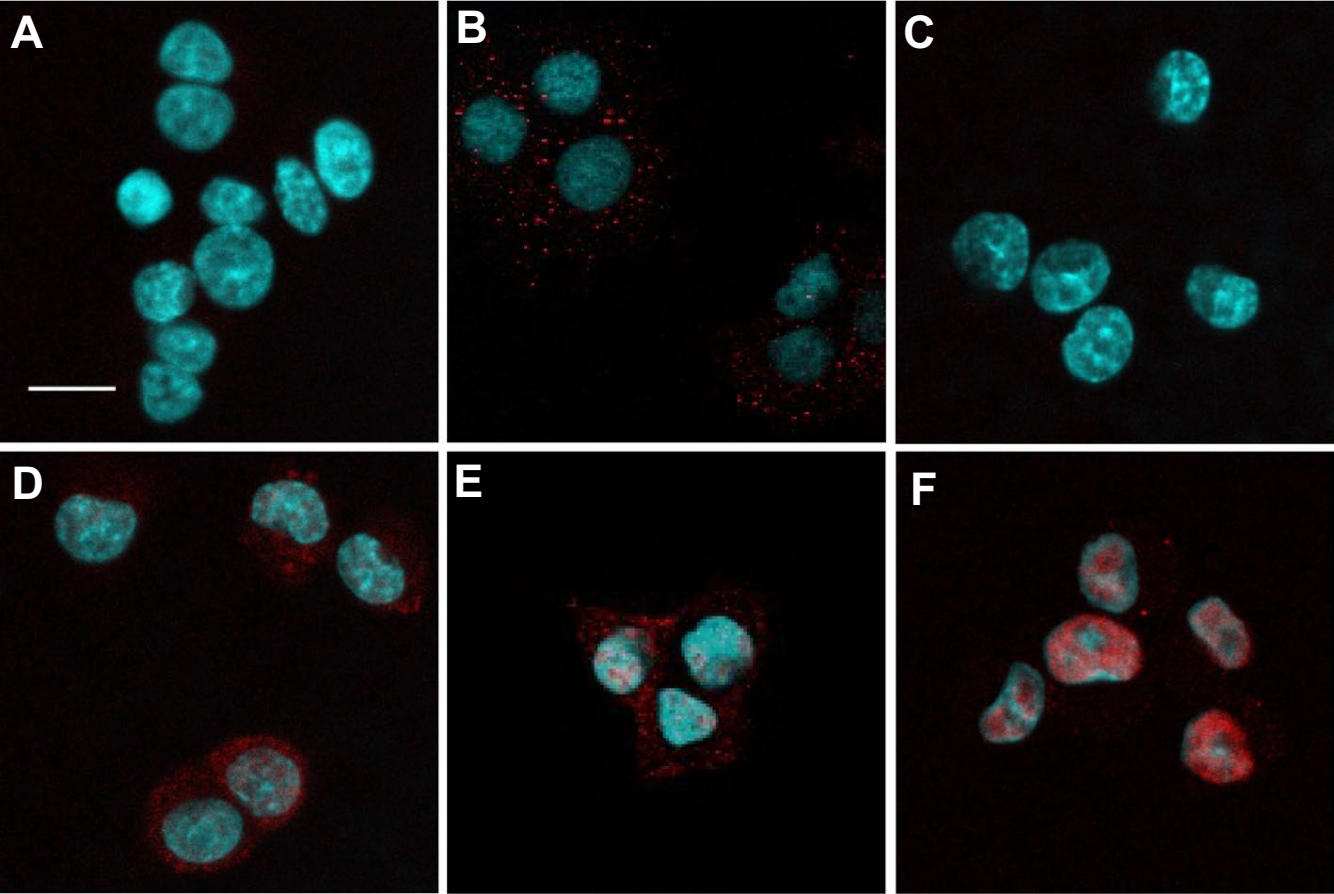
The effect of MG-132 treatment onto the intracellular localization of phosphorylated p38, JNK and c-Jun proteins in PC12 cells by means of confocal fluorescence laser scanning microscopy. Representative microscopic images show p-p38 (**A**,**D**), p-JNK (**B**,**E**) and p-c-Jun (**C**,**F**) signals in untreated (**A**–**C**) or 2.5 µM MG-132-treated [6 h for (**D**) and 24 h for (**E**,**F**)] samples, respectively. The phosphorylated p38, JNK and c-Jun signals are shown in red and nuclei were counterstained with Hoechst 33342 (blue) on all panels. The scalebar on panel (**A**) (10 μm) applies to all (**A–F**) images.

Having seen the phosphorylation changes of p38, JNK and c-Jun we performed immunofluorescence staining targeting the phosphorylated forms of p38, JNK and c-Jun. Using laser scanning confocal fluorescence microscopy no p-p38 and p-c-Jun signal could be detected in untreated PC12 cells (Fig. 3A,C, respectively) while some phosphorylated JNK could be captured in the cytoplasm even without treatment (Fig. 3B). Then we applied MG-132 treatments to provoke maximal activation of the kinases, which meant 6 h for p38 and 24 h for JNK and c-Jun (as shown above under 3.2). After 6 h of MG-132 treatment phosphorylated p38 was predominantly in the cytoplasm of PC12 cells with some additional signal in nuclei (Fig. 3D). After 24 h of MG-132 treatment a strong p-JNK signal was detectable both in the cytoplasm and in nuclei of the cells (Fig. 3E), while in the case of p-c-Jun its markedly increased immunoreactivity was almost exclusively nuclear (Fig. 3F).

### Using kinase-inhibitors to block specific signaling pathways

**Figure 4:**
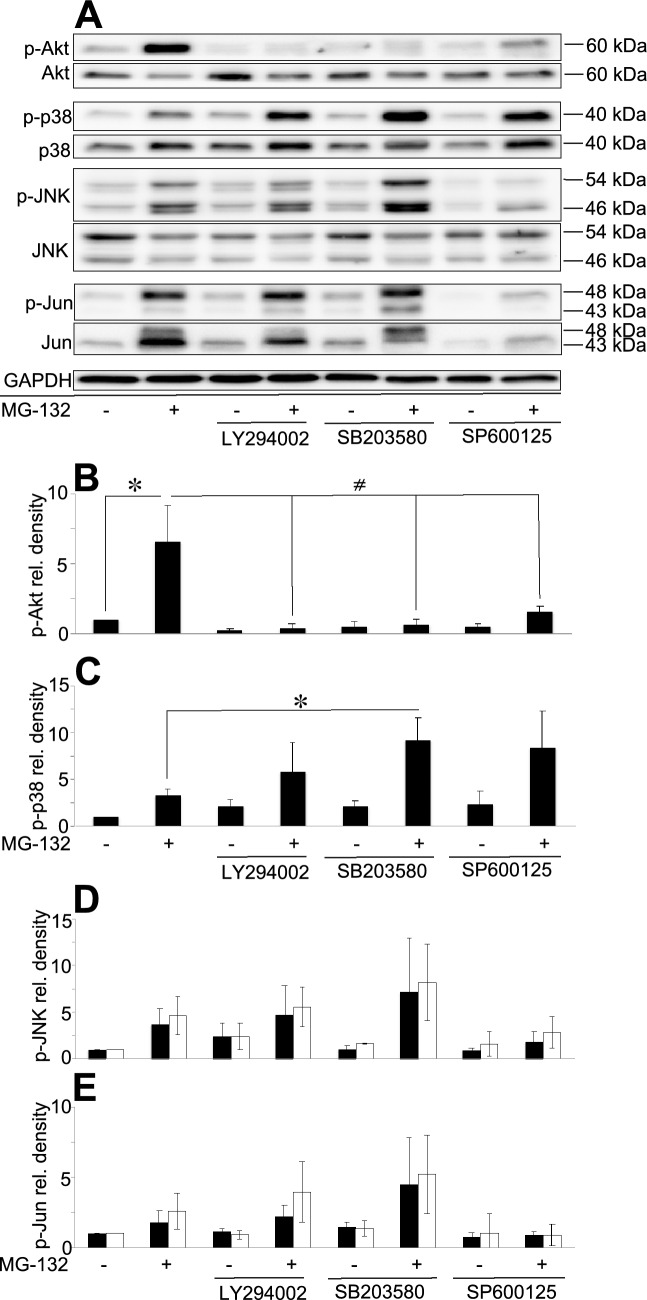
MG-132-induced phosphorylation of various signaling proteins using kinase-inhibitors. (**A**) Immunoblot analysis of Akt, p38, JNK and c-Jun phosphorylation in the presence of the PI3K inhibitor (LY294002) (20 µM), p38 inhibitor (SB203580) (10 µM) or JNK inhibitor (SP600125) (50 µM) in PC12 cells. Cultures were left untreated or were treated for 3 h with MG-132 (2.5 µM) and the inhibitors were added 1 h prior to the proteasome inhibitor treatment and remained present in the cultures until the end of the experiment (altogether 4 h). All blots were cropped, original membrane images are shown in Supplementary Fig. 4. Diagrams show the activation pattern of Akt (**B**), p38 (**C**), JNK (**D**) and c-Jun (**E**). Bars demonstrate the mean ratio ± SD values calculated from the data of three independent experiments. Statistically significant differences are indicated (^*/#^P ≤ 0.05).

In order to specify the role of the above examined kinases further, PC12 cells were cultured 1 h with a kinase inhibitor followed by MG-132 treatment for 3 h. LY294002 is a highly selective inhibitor of phosphatidylinositol 3 kinase (PI3K) that in turn blocks Akt phosphorylation indirectly. SB203580 is a selective inhibitor of p38 MAPK while SP600125 is a potent and selective JNK-1, -2, and -3 inhibitor. Pre-treatment with LY294002 effectively blocked both basal and MG-132-induced Akt phosphorylation (Fig. 4A,B) in PC12 cells but increased (although not significantly) the phosphorylation of p38 (Fig. 4A,C). At the same time it had negligible effects on JNK and c-Jun phosphorylation (Fig. 4A,D,E). Pre-treatment with the p38 inhibitor SB203580 abolished MG-132-induced Akt phosphorylation and also decreased the basal activity of Akt (Fig. 4A,B), but increased the phosphorylation of p38, JNK and c-Jun induced by MG-132 treatment (although the latter two were not significant) (Fig. 4A,C,D,E, respectively). Finally, pre-treatment with the JNK inhibitor SP600125 prevented MG-132-induced Akt, JNK and c-Jun phosphorylation and it also reduced basal Akt activity (Fig. 4A,B,D,E), but increased (although not significantly) both basal and MG-132-induced phosphorylation of p38 (Fig. 4A,C).

We also checked whether the kinase inhibitor treatments affected cell viability. Decreased cell viability was detectable in all samples. The lowest ratio of living cells was measured in samples treated with the combination of LY294002 or SP600125 and MG-132 (56% and 58%, respectively), whereas the SB203580- or SP600125-treated cells showed somewhat higher viability (68% and 66%, respectively) Interestingly, the combination of SB203580 and MG-132 resulted in a similar viability as the MG-132 single treatment (81% and 86%, respectively), while LY294002 treatment alone affected cell viability the least (92%) (Supplementary Fig. 3).

### Phosphorylation of the examined cell signaling molecules in wild type PC12 cells and M-M17-26 mutants upon 3 h of MG-132 treatment.

**Figure 5:**
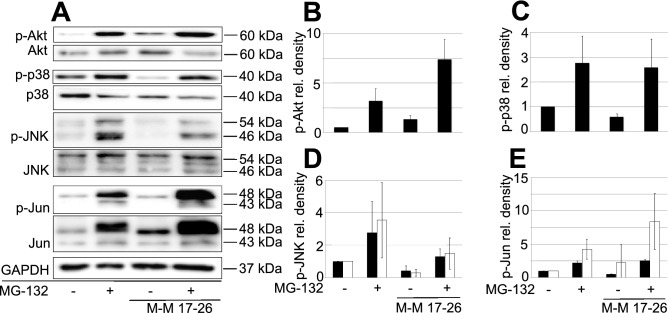
MG-132-induced phosphorylation of the previously examined cell signaling molecules in wild type and dominant negative H-Ras-expressing (M-M17-26) PC12 cell mutants. (**A**) Western blot analysis of Akt, p38, JNK and c-Jun phosphorylation in wild type and M-M17-26 PC12 cells. Cells were treated for 3 h with MG-132 (2.5 µM). All blots were cropped, original membrane images are shown in Suppl. Figure 4. Diagrams show the activation of Akt (**B**), p38 (**C**), JNK (**D**) and c-Jun (**E**) with or without MG-132 treatment. Bars represent the mean ratio ± SD values calculated from data of three independent experiments.

Since Ras is a master regulator of signaling in various cell types, including PC12 cells, we investigated MG-132-induced phosphorylation events in a dominant negative mutant Ras-expressing PC12 cell line (M-M17-26) (Fig. 5). These cells undergo apoptosis earlier upon treatment with MG-132 (our own, unpublished data) compared to wild type PC12 cells and they show no signs of neuronal differentiation in the presence of this proteasome inhibitor. The latter phenomenon can be explained, at least in part, by the impaired ERK1/2 phosphorylation of these mutants^1^. In M-M17-26 cells upon MG-132 treatment we could detect no significant changes in the phosphorylation state of the same examined signaling molecules that we have tested in wild type PC12 cells. Although we could identify elevated p-Akt levels both in the untreated and in the MG-132-treated M-M17-26 cultures (Fig. 5A,B), the basal activity of p38 and JNK seemed to be somewhat lower in M-M17-26 cells (Fig. 5A,C,D), while the MG-132 induced p38 phosphorylation was the same as in wild type cells (Fig. 5A,C). Interestingly, the lack of Ras function decreased JNK and on the other hand increased c-Jun phosphorylation after MG-132 treatment (Fig. 5A,D,E).

## Discussion

Proteasome inhibitors are used to treat hematological malignancies, primarily, due to their apoptosis-inducing potential. In case of the agent Bortezomib, for example, four cellular processes were identified behind its apoptosis-inducing ability: (1) it inhibits the NFκB pathway, (2) it directly induces apoptosis via JNK and p53, (3) it prevents the degradation of pro-apoptotic proteins (like that of Bim, Bid, Bik, NOXA) and (4) it provokes endoplasmic reticulum (ER) stress and unfolded protein response (UPR)^17^.

From this point of view it was an interesting observation that the proteasome inhibitor MG-132 treatment induced neuronal differentiation in PC12 cells^18^, an effect mediated by sustained ERK1/2 phosphorylation and nuclear translocation of active ERK1/2^1^. The neuronal differentiation-inducing effect of MG-132, however, is only transient (up to the first 24 h). Upon prolonged (more than 24 h) exposure of PC12 cells to MG-132, events of differentiation are rapidly taken over by cellular deterioration, apparently as a result of apoptosis. A microarray analysis has also made it evident that proteasome inhibition with lactacystine activates neuroprotective and pro-apoptotic pathways as well^19^.

In the present study we examined the molecular background of the above biphasic response elicited by the proteasome inhibitor MG-132 in PC12 cells. Upon MG-132 treatment up to 24 h we could observe prolonged activation of Akt -the kinase mediating cell survival- and that of stress-mediating signaling components (p38, JNK, c-Jun). Around 24 h of MG-132 treatment ERK1/2^1^ and Akt activation already declined and the signs of apoptosis were increasingly detectable in the cultures. After 24 h the stress signaling molecules p38, JNK and c-Jun were still highly active. Most importantly, in samples treated for 24 h with MG-132 the activating cleavage of caspase-3 was also evident. The results of Wu and colleagues^20^ have previously confirmed that sustained -but not transient- activation of JNK contributes to apoptosis, which is in line with our above observation of sustained JNK phosphorylation upon MG-132 treatment in PC12 cells before and as they underwent apoptosis.

In addition, we examined the localization of activated stress signaling molecules: the MAPKs (p38 and JNK) were detected both in the cytoplasm and in the nucleus, while the transcription factor p-c-Jun was observed exclusively in nuclei of MG-132-treated PC12 cells.

An important point regarding mechanism of action is how proteasome inhibitor treatment can increase the expression level or activation of signaling molecules? In general, it can either prevent proteasomal degradation of the phosphorylated/active form of a signaling molecule itself and/or that of its upstream activator(s). All signaling molecules that we examined in this study (and even additional molecules involved in their activation or inactivation here not examined) can be broken down by the proteasome. Our current results indicate that the phosphorylated forms of Akt, p38, JNK and c-Jun are targets of regulation exerted by the proteasome in PC12 cells. The sustained blockade of the proteasome has led to the accumulation of activated p38, JNK and caspase-3, which, in turn, triggered apoptosis. Both p38 and JNK are involved in the activation of pro-apoptotic Bcl-2 family members (like Bax and Bim) and also in the inactivation of anti-apoptotic Bcl-2 isoforms (Bcl-2 itself, for example), eventually leading to cytochrome c release and caspase activation^21^.

Long term proteasome inhibitor-induced PC12 cell apoptosis demonstrated in our study could add to our understanding of the neurological side effects observable in proteasome inhibitor-treated patients. In some neurodegenerative diseases, like Parkinson’s disease, a decreased function of the proteasome was observed resulting in the accumulation of mis-folded proteins and leading to neuronal degeneration^22^. The first proteasome inhibitor approved by the FDA in 2003 for the treatment of multiple myeloma was Bortezomib^23^ that belongs to the group of peptide-boronates. Carfilzomib is an epoxyketon type proteasome inhibitor that has acquired FDA approval in 2012^24^, while Ixazomib -which belongs to the same group as Bortezomib- was the first orally administered proteasome inhibitor approved in 2015 for the treatment of relapsed and refractory multiple myeloma^25^, just like Carfilzomib. Bortezomib-induced peripheral neuropathy (BIPN), a well-documented complication, develops in 30–60% of treated patients^26^. Earlier studies identified ER stress, mitochondrial dysfunction and trafficking problems, inflammatory response, DNA damage and microtubule-related changes as additional components in the background of BIPN^26^. Based on our results neuronal apoptosis could also contribute to the development of neuropathy, however, further studies are needed to directly confirm this hypothesis.

**Figure 6:**
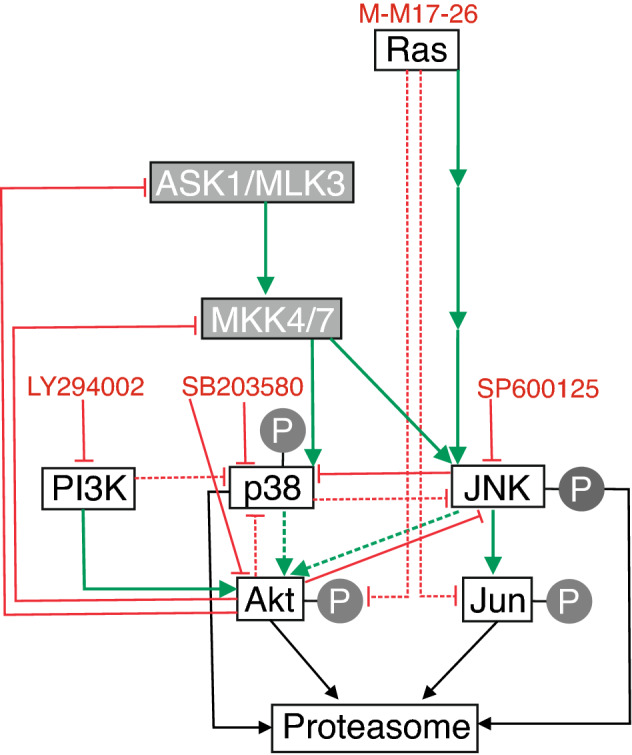
Proposed summary of proteasome inhibitor- and kinase inhibitor-induced signaling in PC12 cells. Activation is indicated by green arrows, inhibition by red lines. Dashed lines represent proposed connections based on our presented results, continuous lines show already confirmed connections. Signaling molecules studied in this work are indicated in white boxes with black captions, other components not studied by us are in grey boxes with white captions.

Finally, by combining proteasome inhibition with kinase inhibitor treatments we could interfere with potentially critical connections between Akt, p38, JNK and c-Jun. We summarize our currently proposed network of these interactions based on our presented experimental data and on additional ones from the literature in Fig. 6.

As expected, the PI3K inhibitor LY294002 completely abolished Akt activation (Fig. 6). Interestingly, the inhibition of PI3K enhanced MG-132-induced p38 phosphorylation, suggesting that PI3K or Akt normally has an inhibitory effect on p38 activation (Fig. 6). Other studies have reported, for example, that Akt inhibits MKK4 (MAPK Kinase 4), ASK1 (Apoptosis Signal-regulated Kinase 1) and MLK3 (Mixed Lineage Kinase 3)^27^, which kinases are the upstream activators of p38 and JNK as well. Although it has been documented that Akt has an inhibitory effect on JNK through upstream kinases^27^, we could not detect a significant elevation of JNK- and c-Jun-activation upon the double treatment of LY294002 and MG-132, compared to proteasome inhibitor treatment alone (Fig. 6).

The ATP competitive p38 inhibitor, SB203580 on the other hand had a negative effect on Akt phosphorylation, suggesting that p38 plays a role in MG-132-induced Akt activation in PC12 cells (Fig. 6). The direct inhibition of Akt by SB203580 has also been reported^28^. SB203580 binds the ATP binding site of p38 and hence, it can inhibit the kinase activity, but not the phosphorylation of the enzyme by upstream kinases. We observed significantly elevated p38 phosphorylation after combined SB203580 and MG-132 treatment compared to the sample treated with MG-132 alone. MG-132-induced JNK and c-Jun phosphorylations were also elevated upon SB203580 pretreatment, a phenomenon that can have several explanations (Fig. 6): (1) either p38 itself inhibits JNK phosphorylation (Fig. 6), or (2) Akt can inhibit JNK activation at various levels [e.g. at ASK1, MLK3 (MAPKKKs), MKK4/7 (MAPKK) or JNK] and when Akt is inactivated by SB203580 directly or indirectly through p38 inactivation, Akt is not able to inhibit the JNK pathway (Fig. 6). (3) Furthermore, it has been shown that SB203580 increases JNK phosphorylation through the activation of MLK3, the MAPKKK involved in JNK activation^29^. Finally, it is worth mentioning that SB203580-induced MLK3 phosphorylation can be a result of the previously mentioned Akt inactivation and the consequentially impaired Akt-induced MLK3 inhibition.

SP600125 is an ATP competitive pan-JNK inhibitor, that decreased the MG-132-induced Akt phosphorylation significantly, suggesting that the activity of JNK is required for MG-132-induced Akt activation in PC12 cells (Fig. 6). The MG-132 induced p38 phosphorylation was elevated upon JNK inhibitor pretreatment, indicating that JNK has a—direct or indirect—inhibitory effect on p38 activation (Fig. 6). For example JNK activates an E3 ubiquitin ligase, Itch, that is involved in MKK4 inactivation^30^ and when this inhibitory pathway is blocked by SP600125, MKK4 can activate p38. Decreased c-Jun phosphorylation shows that JNK activity is blocked by SP600125 (Fig. 6), but JNK phosphorylation was also diminished, suggesting that there could either be a positive feedback loop in this pathway or autophosphorylation of JNK itself is responsible for it (Fig. 6). Fey and colleagues suggested a positive feedback in JNK signaling, through which JNK can activate its upstream kinases ASK and MLK^27^. Furthermore, JNK2 autophosphorylation has been reported too^31^. In addition to these two abovementioned processes, there are other possibilities to explain how SP600125 pretreatment could decrease JNK phosphorylation (Fig. 6). Based on the previous conclusions that SP600125 increases p38 activity and p38 activates Akt, which in turn inactivates the upstream kinases of JNK (ASK/MLK, MKK4/7), this whole process could lead to decreased JNK phosphorylation as well (Fig. 6).

In the absence of functioning Ras, proteasome inhibitor treatment had substantially different consequences. In dominant negative Ras-mutant M-M17-26 PC12 cells MG-132-induced ERK phosphorylation is completely missing^1,12^ and neuronal differentiation of the cells is absent too (our own, unpublished data). However, even these mutant cells exhibit the signs of apoptosis after prolonged MG-132 treatment (in fact somewhat sooner than normal PC12 cells, data not shown). This observation supports that the functionality of the Ras-ERK pathway is required to experience the effects of MG-132 treatment’s early phase, i.e. neuronal differentiation in PC12 cells. The later apoptosis-inducing events, however, remained unaffected by Ras inhibition.

In conclusion, we demonstrated the apoptosis-inducing ability of MG-132 in PC12 cells and identified that the phenomenon of programmed cell death is coupled to a gradual shift in MG-132-induced signaling towards increased activity of stress kinases (JNK and p38) and caspase-3 from the initial stimulation of pro-differentiation/survival molecules (ERK1/2 and Akt). Furthermore, we propose a complex regulatory network among signaling components like Akt, JNK and p38. Finally, we showed that some kinase inhibitors could potentiate the effects of the proteasome inhibitor MG-132. The latter observation and the proposed regulatory connections between the examined signaling components could even contribute to the establishment of novel therapeutic combinations in the future.

## Supplementary Information

Supplementary Information 1.

Supplementary Information 2.

## Abbreviations

Akt: A protein kinase, also known as Protein Kinase B (PKB)
ASK1: Apoptosis Signal-regulated Kinase 1, it is a MAPKKK that can activate the JNK pathway
Bcl-2: B-cell lymphoma 2, an antiapoptotic protein
Bax: Bcl-2-associated X, a proapoptotic protein
Bid: BH3 interacting domain death agonist, a proapoptotic protein
Bik: Bcl-2 interacting killer, a proapoptotic protein
Bim: Bcl-2 interacting mediator of cell death, a proapoptotic protein
BIPN: Bortezomib-induced peripheral neuropathy
c-Jun: A transcription factor, a substrate of JNK
Cy3: A fluorophore cyanine dye
DMEM: Dulbecco's Modified Eagle's Medium
DMSO: Dimethyl sulfoxide
DTT: Dithiothreitol
ECL: Enhanced chemiluminescence
EDTA: Ethylene diamine tetraacetic acid
EGTA: Ethylene glycol tetraacetic acid
ER: Endoplasmic reticulum
ERK1/2: Extracellular signal-regulated kinase 1 and 2
E3: A ubiquitin ligase enzyme
FACS: Fluorescence-activated cell sorter
FITC: Fluorescein isothiocyanate
FL1/FL2: Fluorescence channel 1/2
GAPDH: Glyceraldehyde 3-phosphate dehydrogenase
HRP: Horseradish-peroxidase
Itch: A HECT domain-containing Nedd4-like ubiquitin protein ligase, it can be activated by JNK
JNK: C-Jun N-terminal kinase, also known as stress-activated protein kinase (SAPK)
LY294002: A highly selective inhibitor of phosphatidylinositol 3 kinase (PI3K)
MAPK: Mitogen-activated protein kinase
MAPKK: MAPK kinase
MAPKKK: MAPKK kinase
MEK: Mitogen-activated protein kinase/ERK kinase, it is a MAPKK
MG-132: A peptidyl-aldehyde type proteasome inhibitor
MKK4: MAPK kinase 4, it can activate JNK and/or p38 MAPK
MKK4/7: MAPK kinase 4/7
MLK3: Mixed lineage kinase 3, it is a MAPKKK, it can activate the JNK and/or p38 MAPK pathway(s)
M-M17-26: Dominant negative H-Ras protein-expressing PC12 cell line
NFκB: Nuclear factor κ B
NGF: Nerve growth factor
NOXA: Phorbol-12-myristate-13-acetate-induced protein 1, a proapoptotic protein
p38 MAPK: A MAP kinase with the molecular weight of 38 kDa
PBS: Phosphate buffered saline
PC12: Rat pheochromocytoma cell line
PI: Propidium-iodide
PI3K: Phosphatidylinositol 3 kinase
PMSF: Phenyl methane sulfonyl fluoride
PVDF: Polyvinylidene difluoride
p53: A tumor suppressor protein with the molecular weight of 53 kDa
Ras: A monomeric G-protein, it can activate the ERK pathway
S: Centrifugal sedimentation constant, named after Svedberg
SAPK/JNK: Stress-activated protein kinase, also known as JNK
SB203580: An ATP-competitive p38 MAPK inhibitor
SDS: Sodium dodecyl sulfate
SP600125: An ATP-competitive pan-JNK inhibitor
TBS: Tris-buffered saline
TBS-T: 0.1% Triton X-100 dissolved in TBS
TBS-Tween: 0.2% Tween-20 dissolved in TBS
UPS: Ubiquitin–proteasome system
WST-1: A tetrazolium salt used to determine the ratio of living cells
