# Supplementary material for: Prolonged treatment with the proteasome inhibitor MG-132 induces apoptosis in PC12 rat pheochromocytoma cells
Source: Sci Rep. 2022 Apr 6;12:5808. doi: 10.1038/s41598-022-09763-z (PMC8987075; doi:10.1038/s41598-022-09763-z)

Suppl. Fig. 4 (Tarjanyi et al. original Western-blots)

Fig. 2 p-Akt

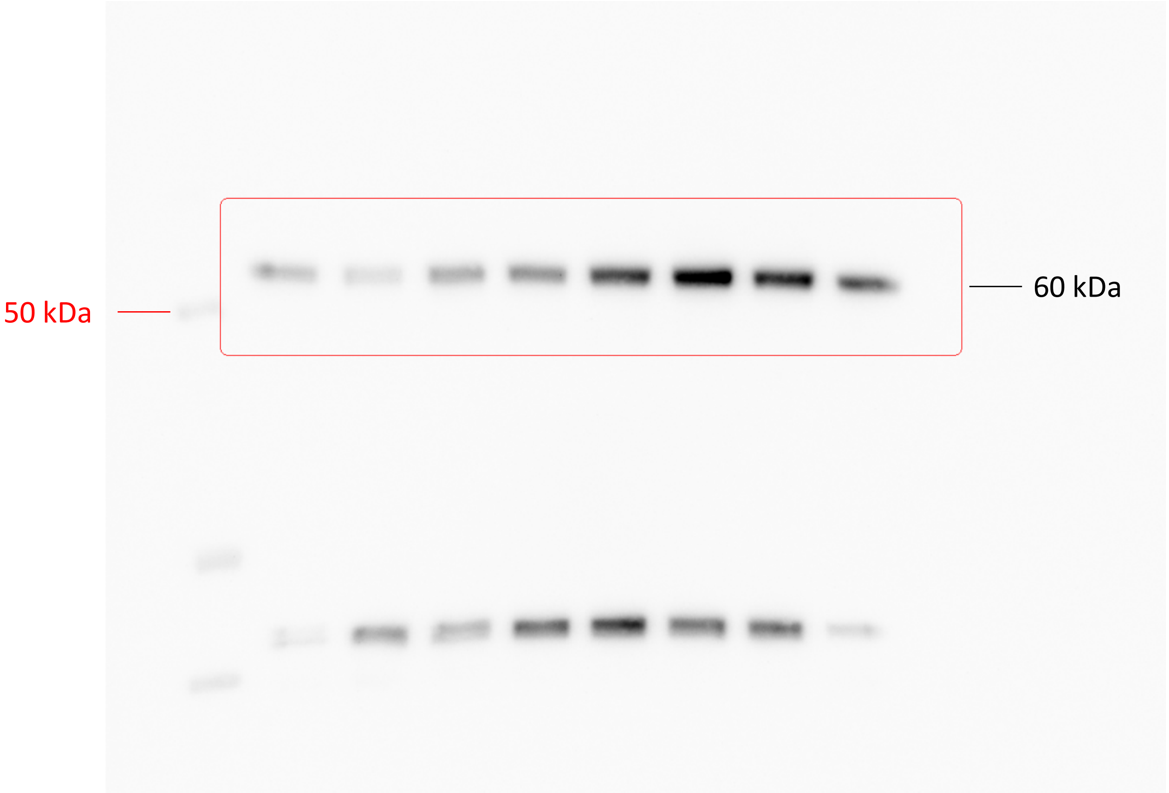

Fig. 2 Akt

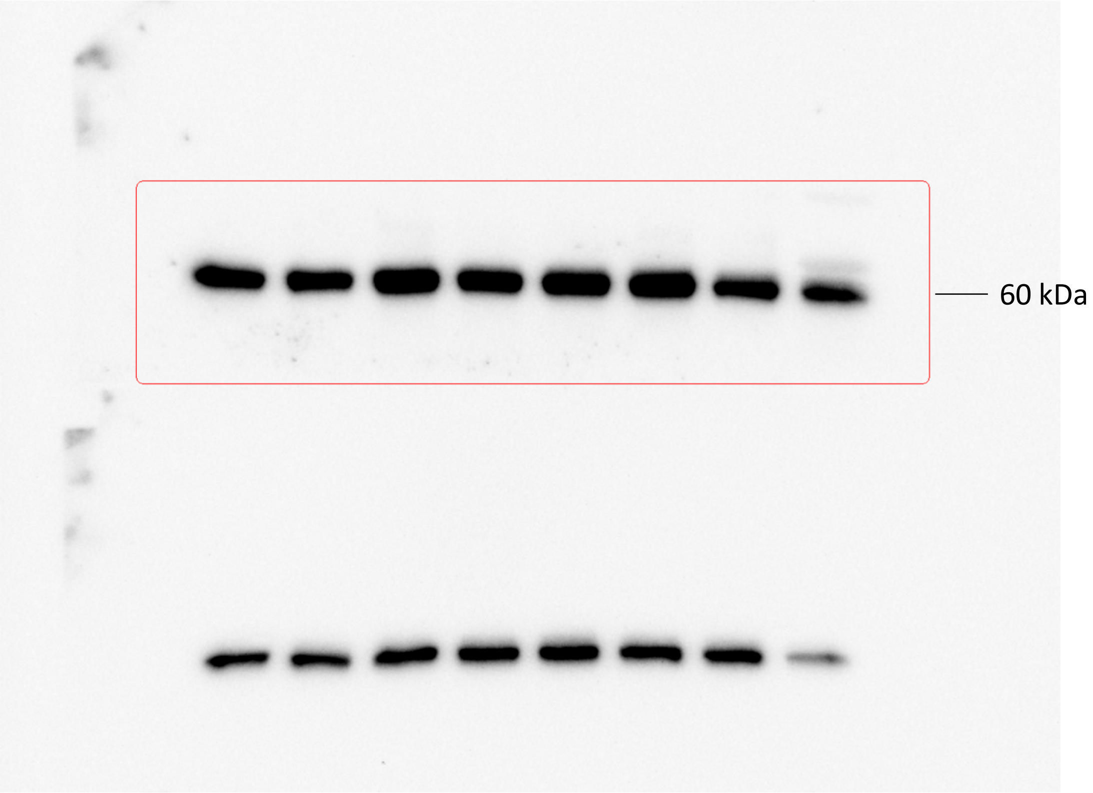

Fig. 2 p-p38

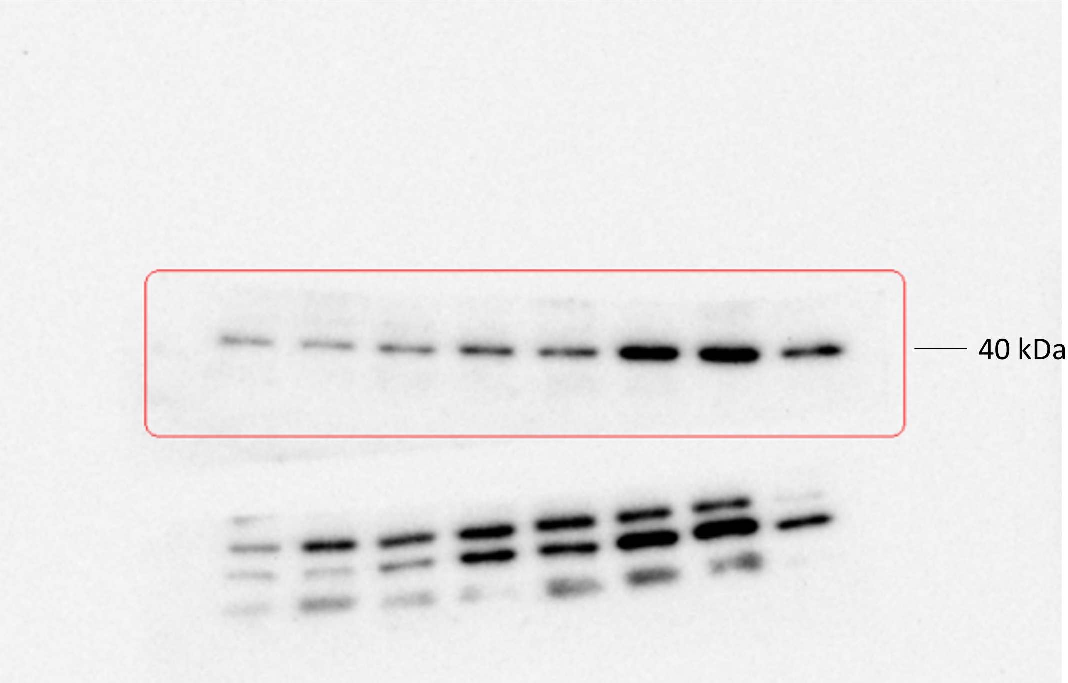

Fig. 2 p38

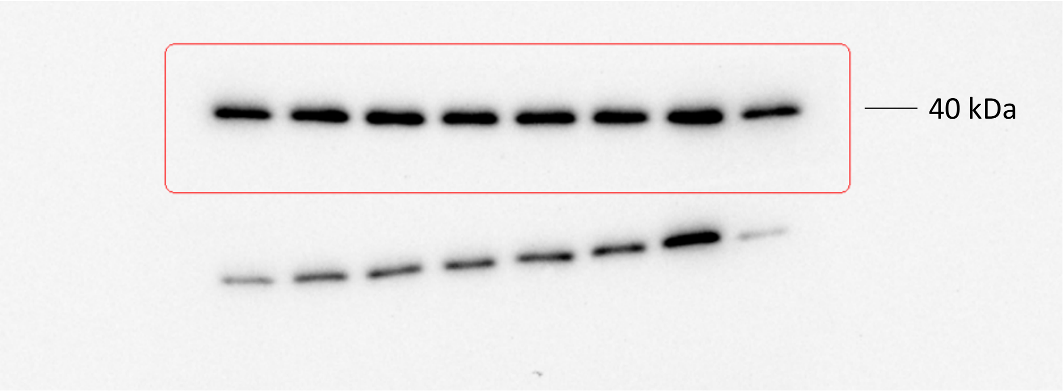

Fig. 2 p-JNK

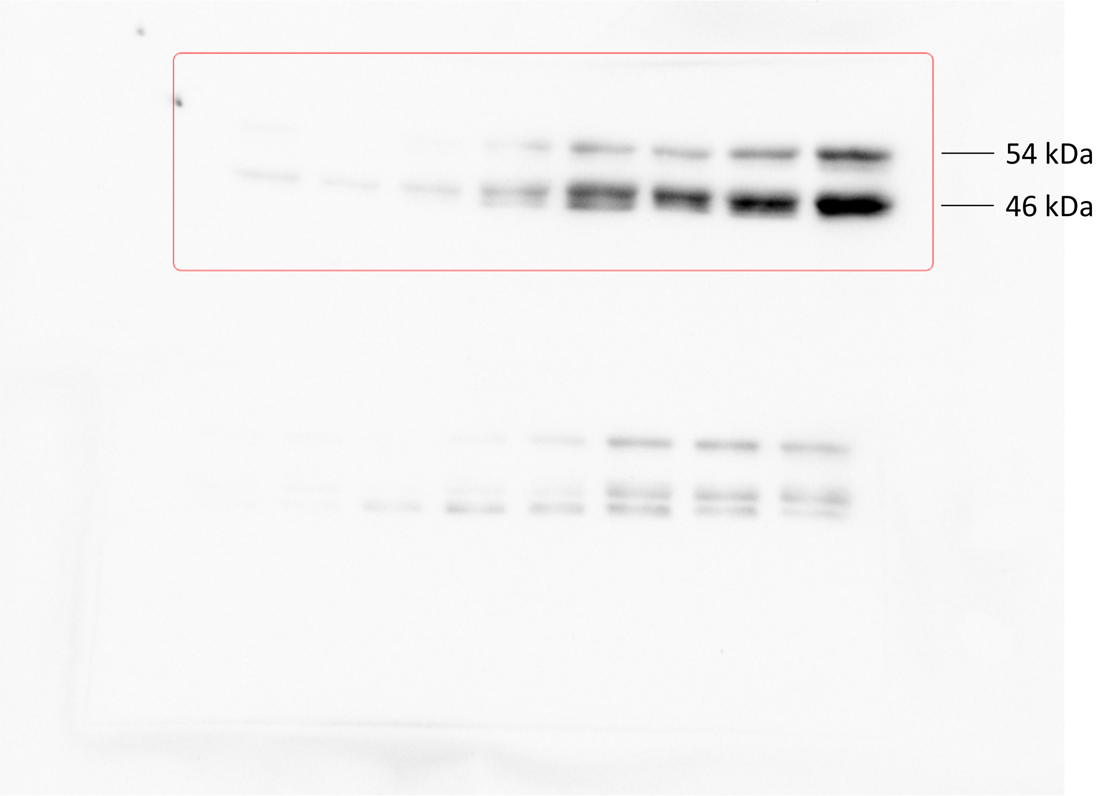

Fig. 2 JNK

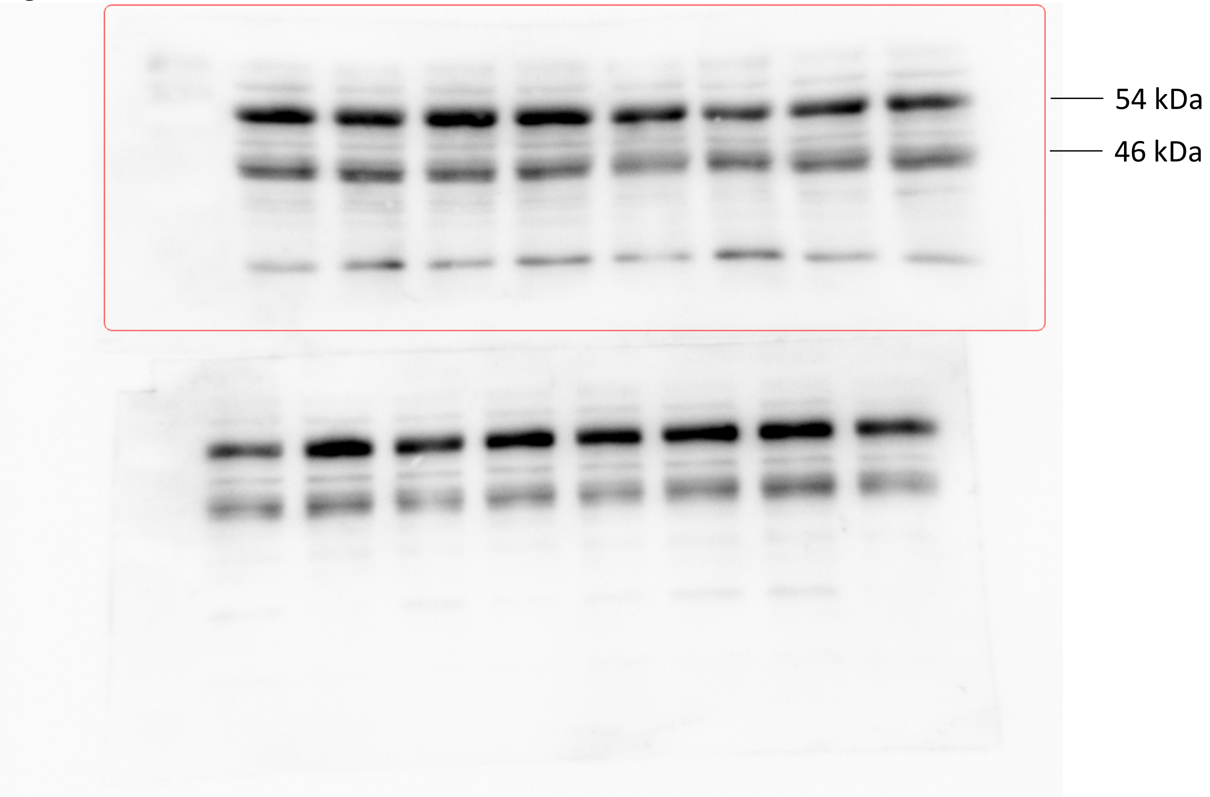

Fig. 2 p-Jun

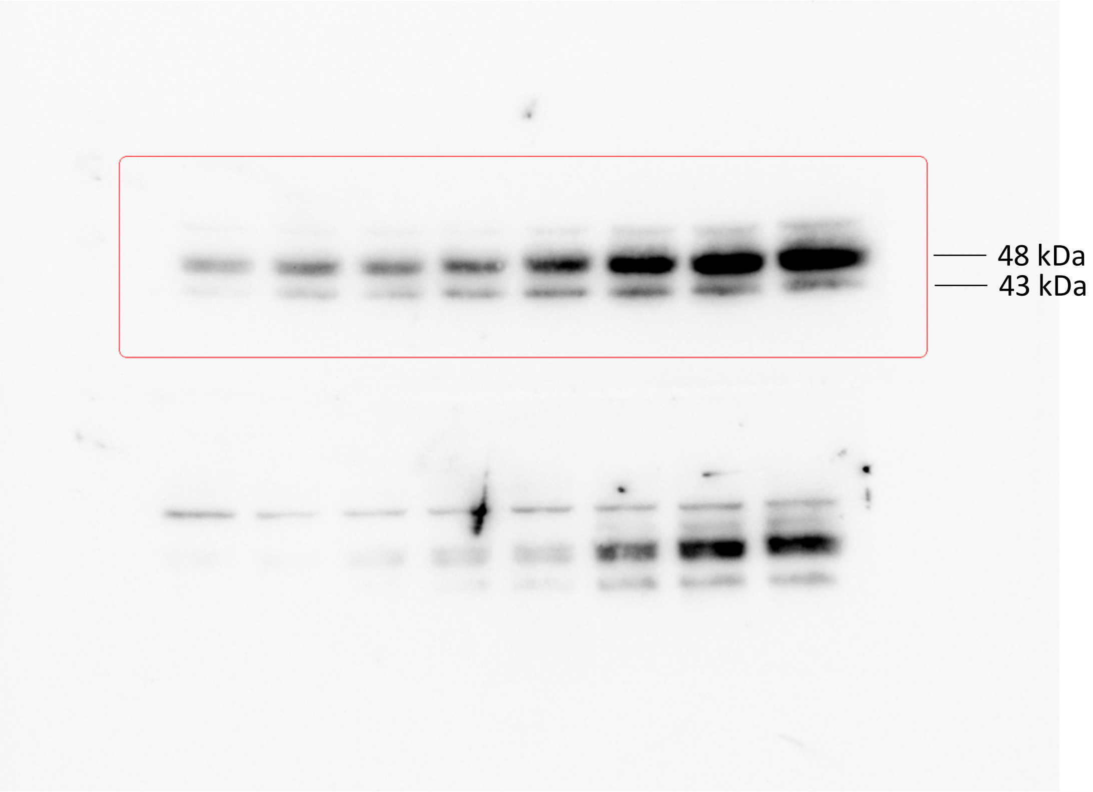

Fig. 2 Jun

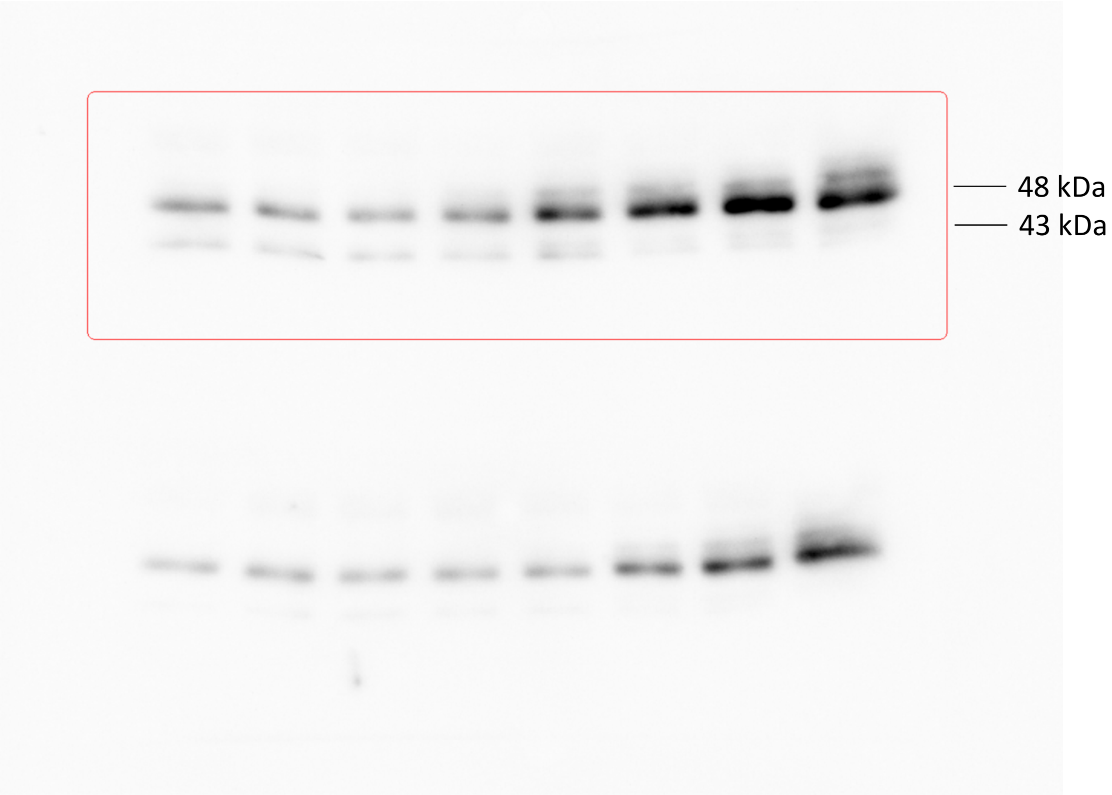

Fig. 2 Cleaved Casp-3

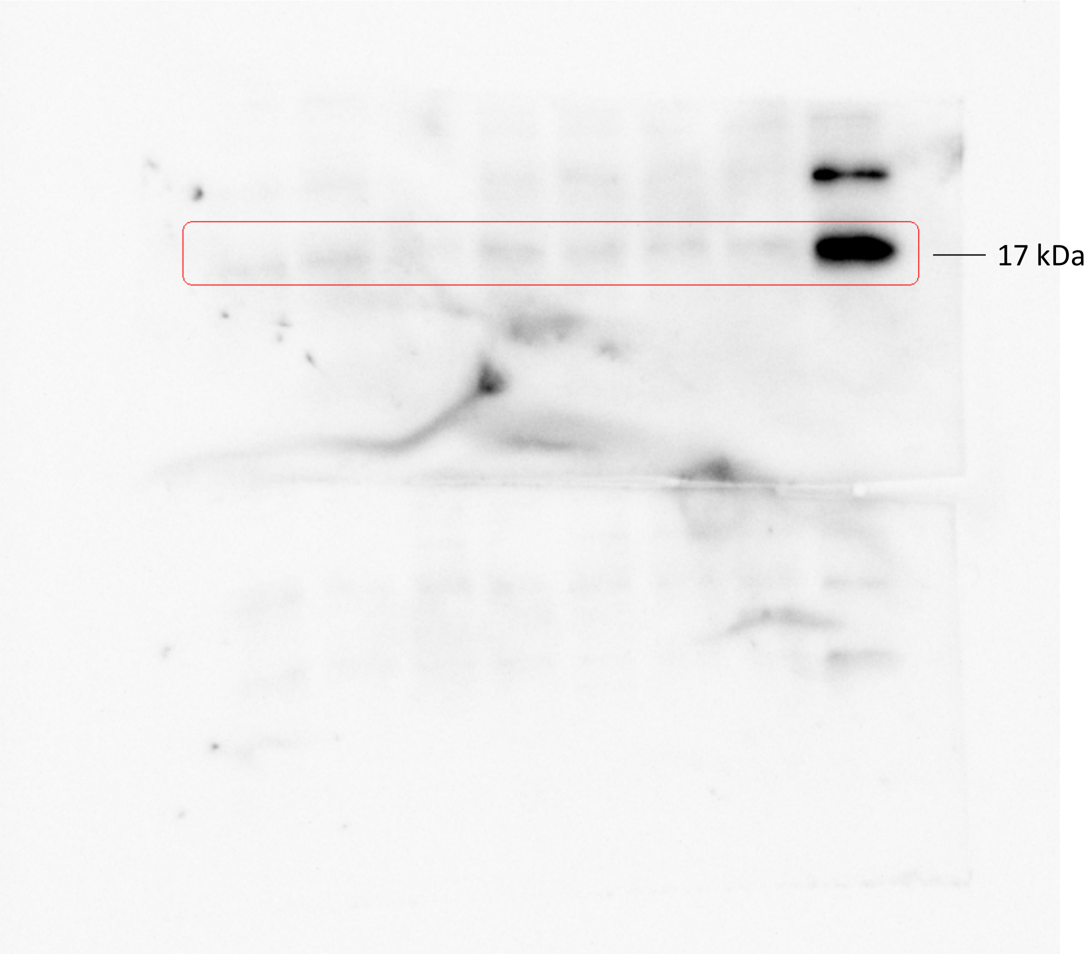

Fig. 2 GAPDH

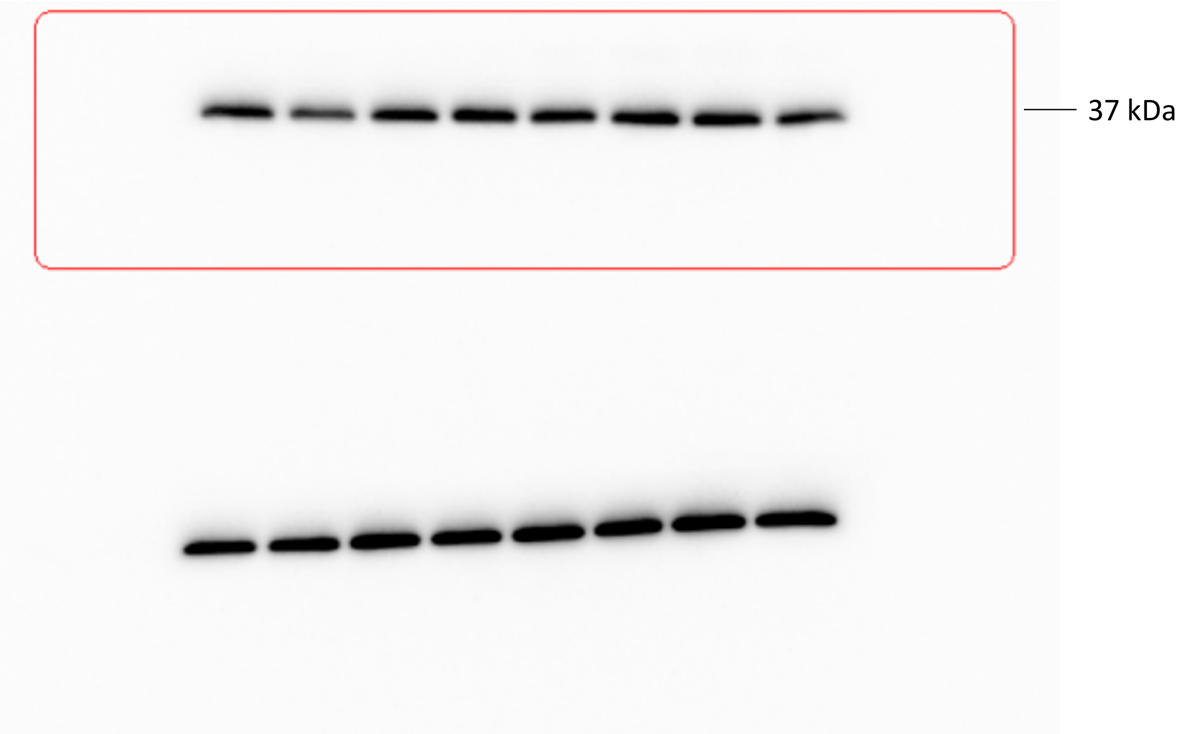

Fig. 4 p-Akt

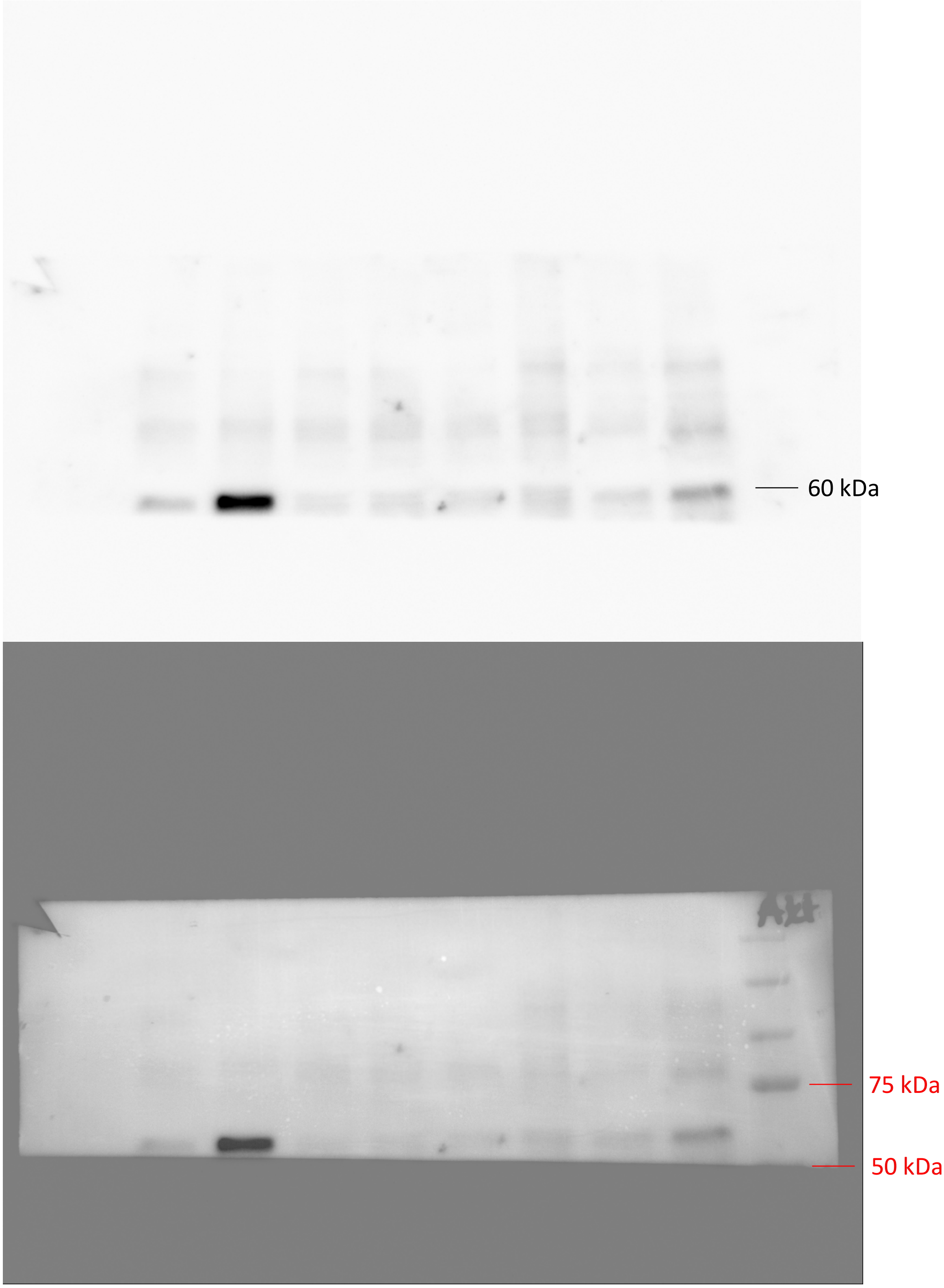

60 kDa

75 kDa

50 kDa

Fig. 4 Akt

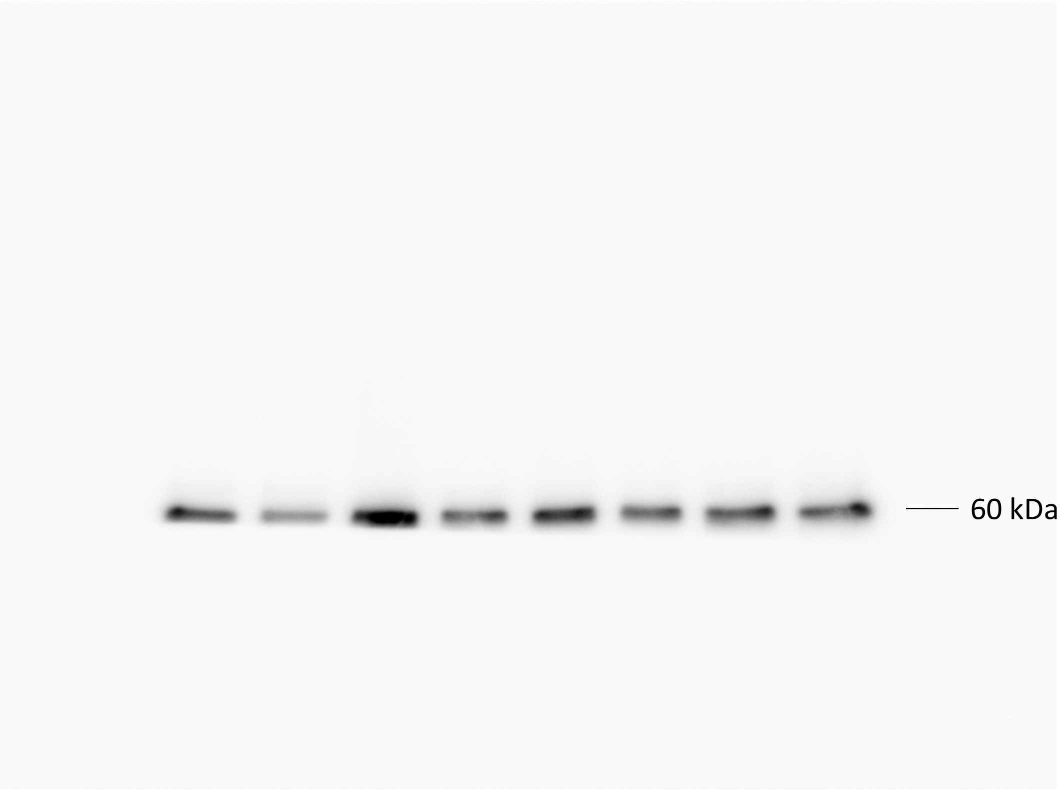

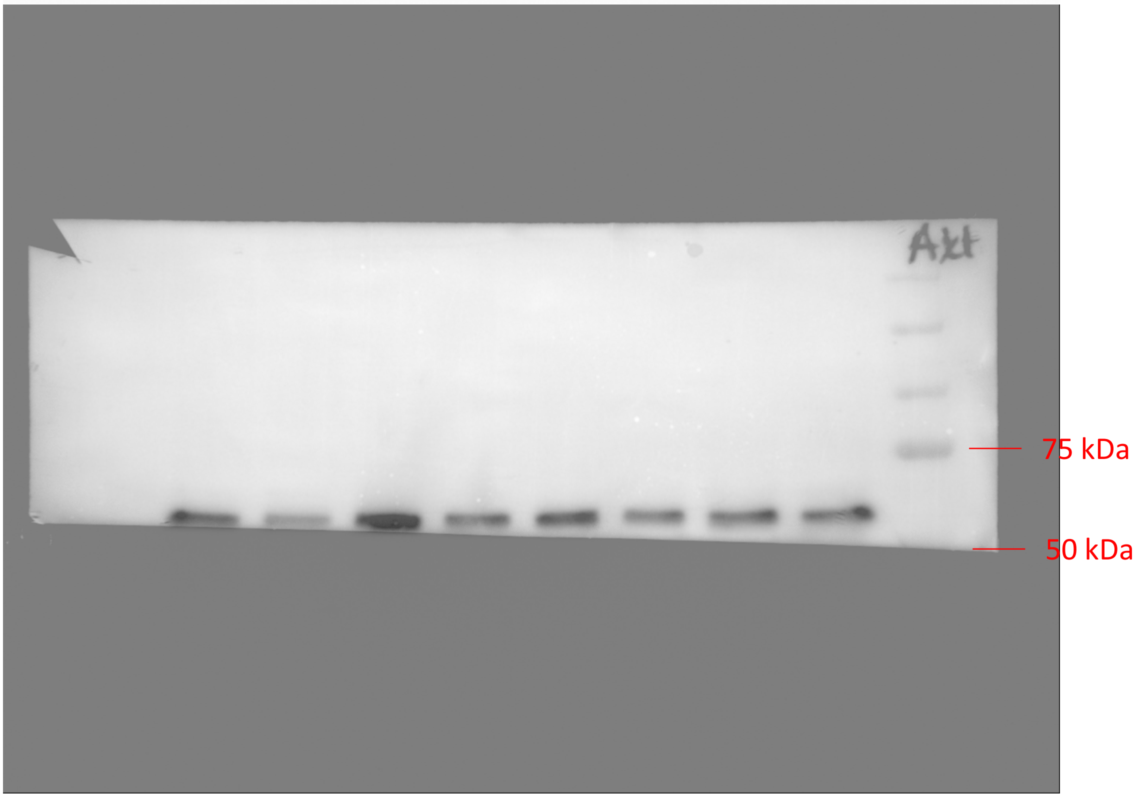

Fig. 4 p-p38

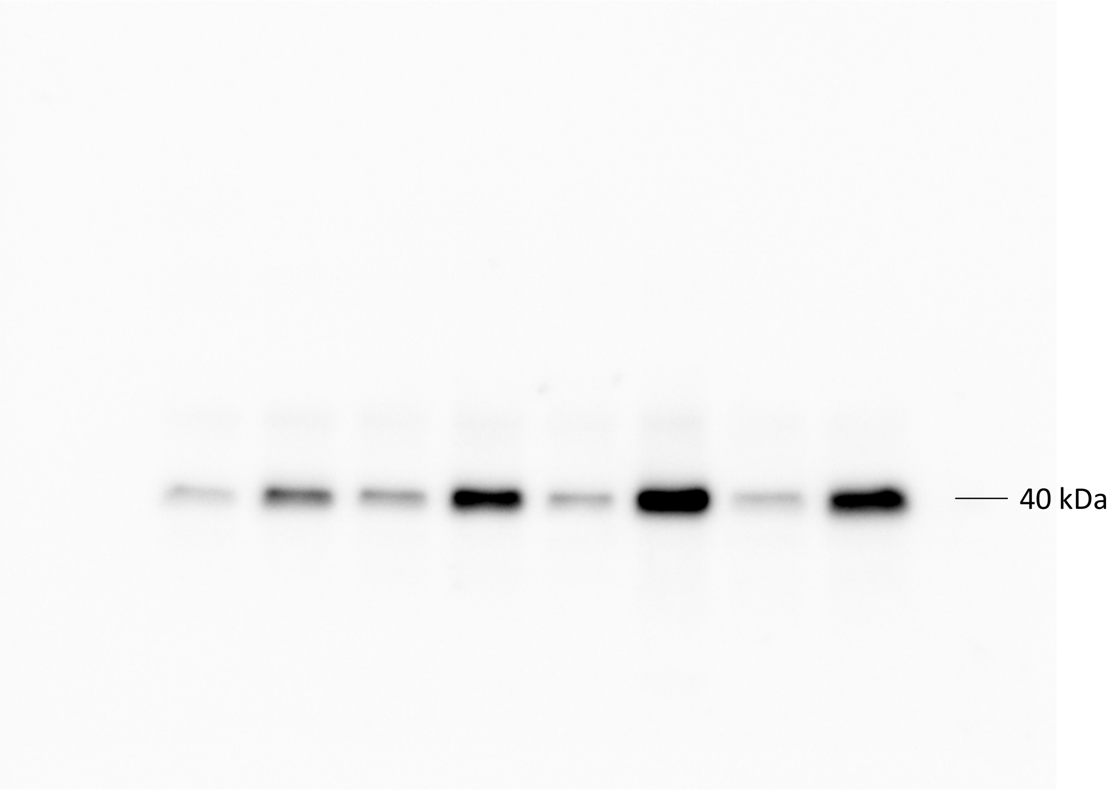

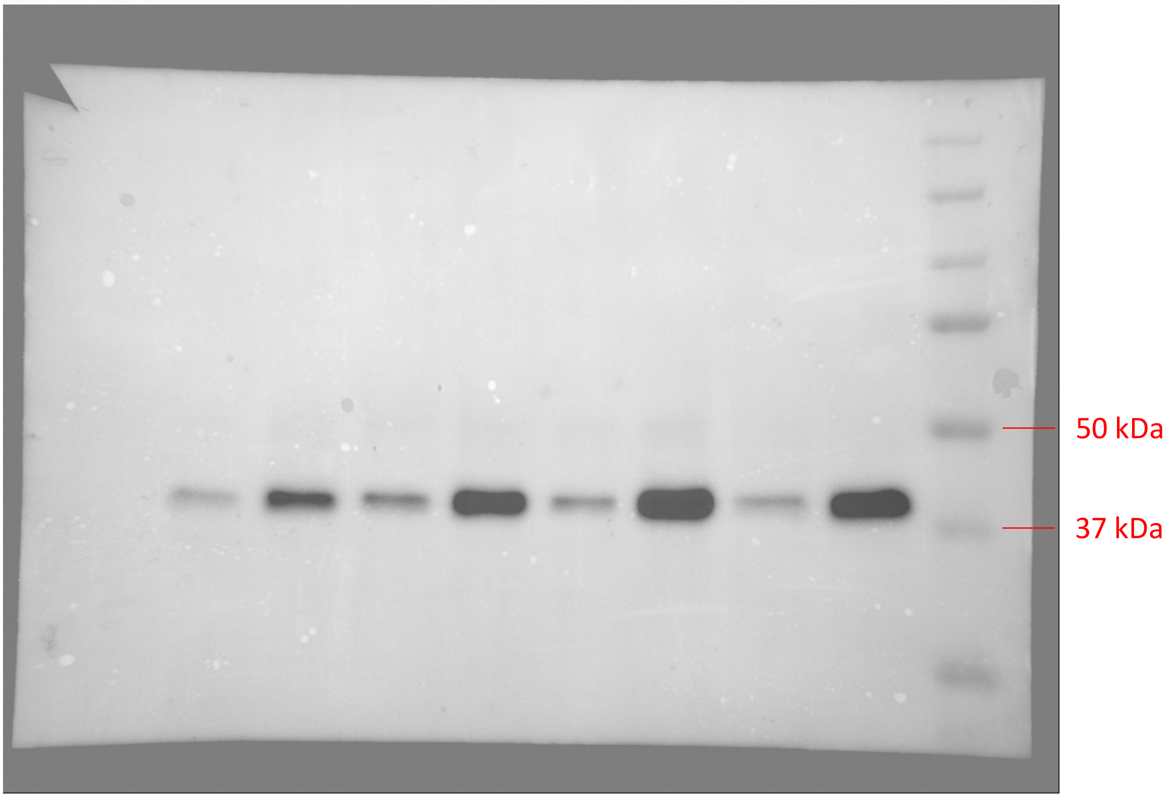

Fig. 4 p38

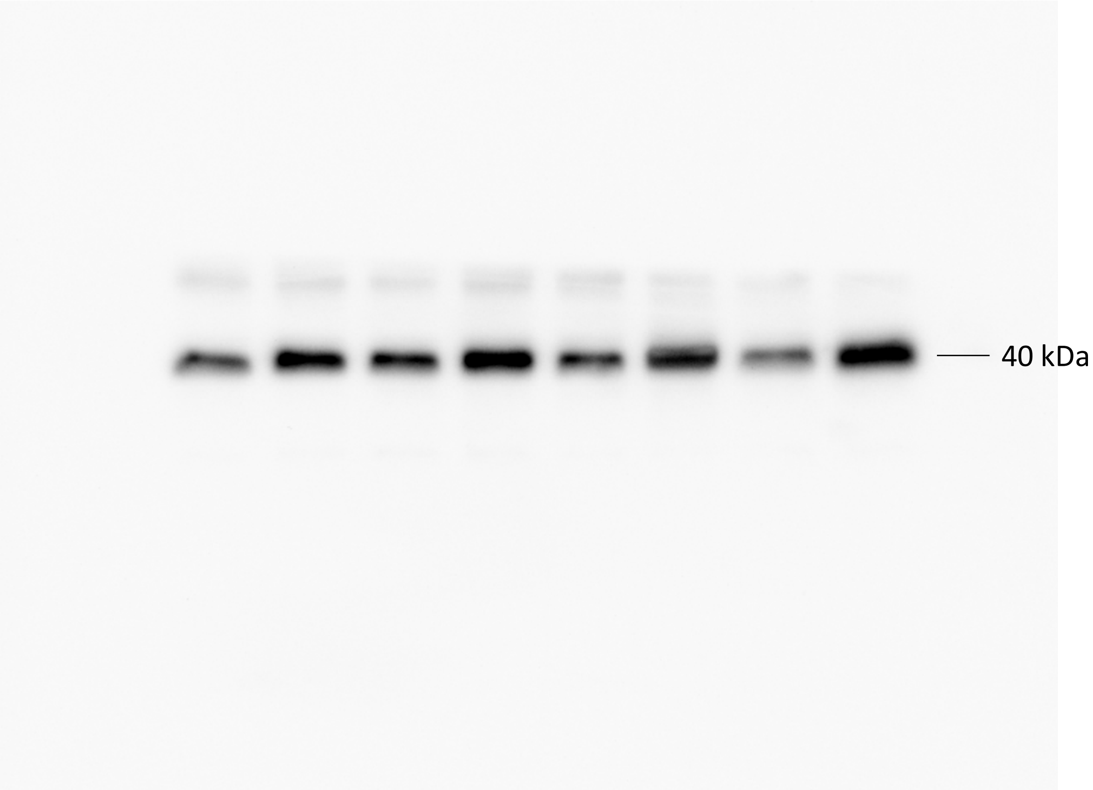

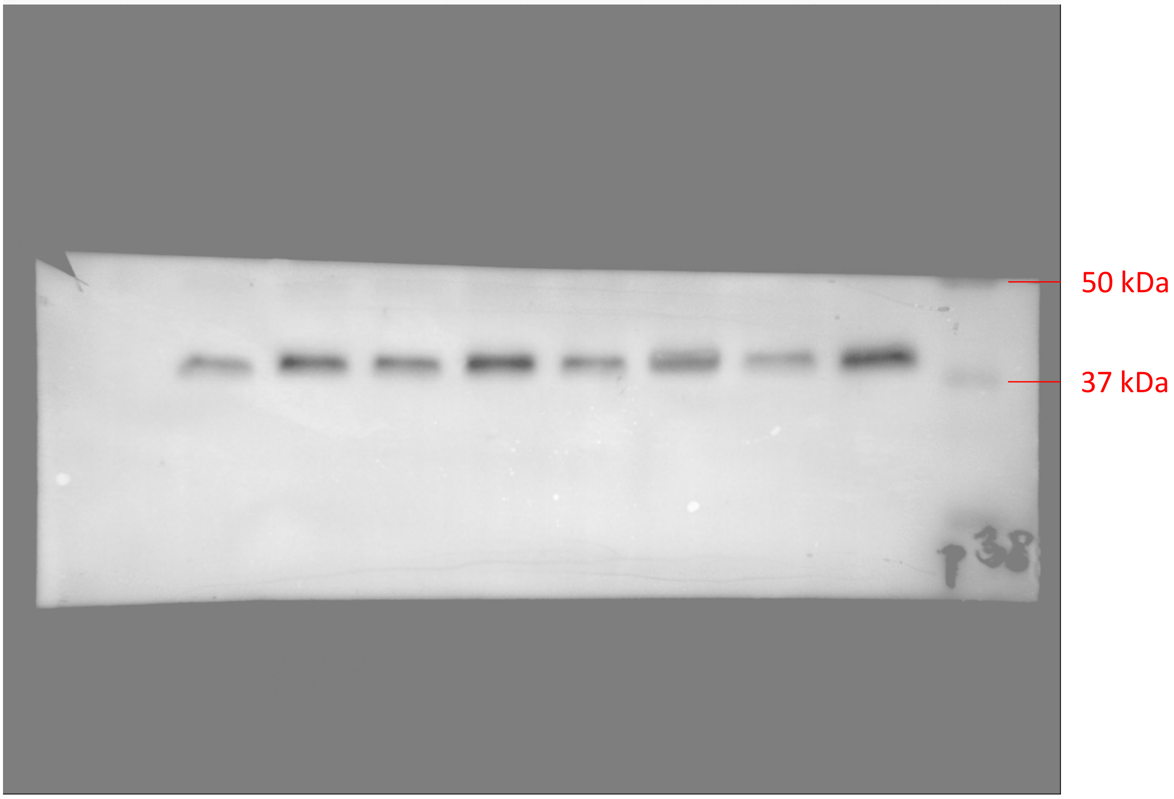

Fig. 4 p-JNK

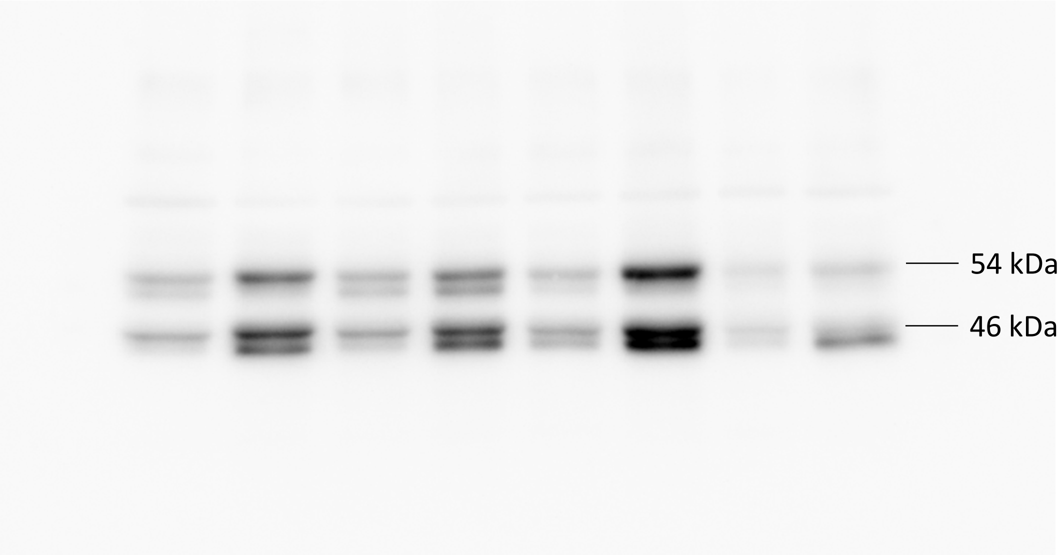

Fig. 4 JNK

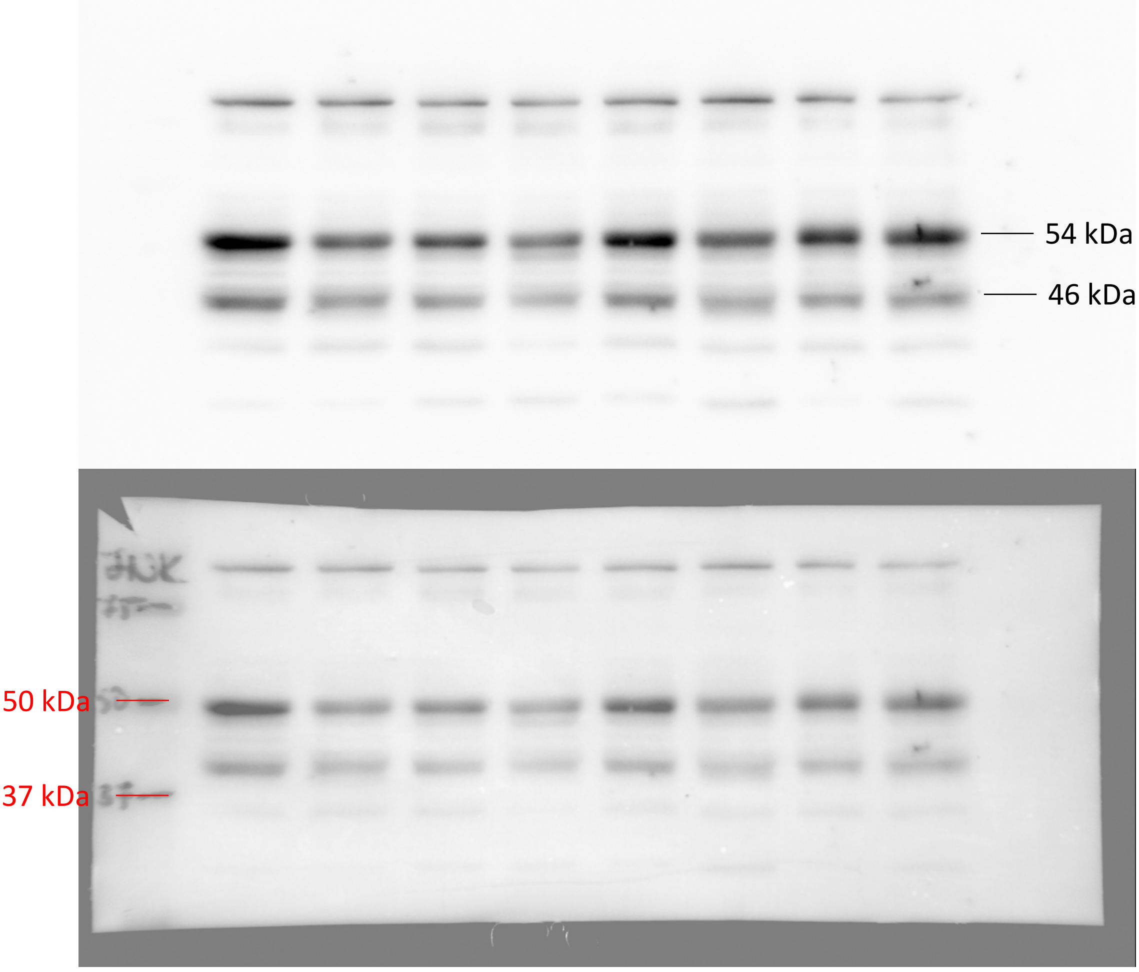

Fig. 4 p-Jun

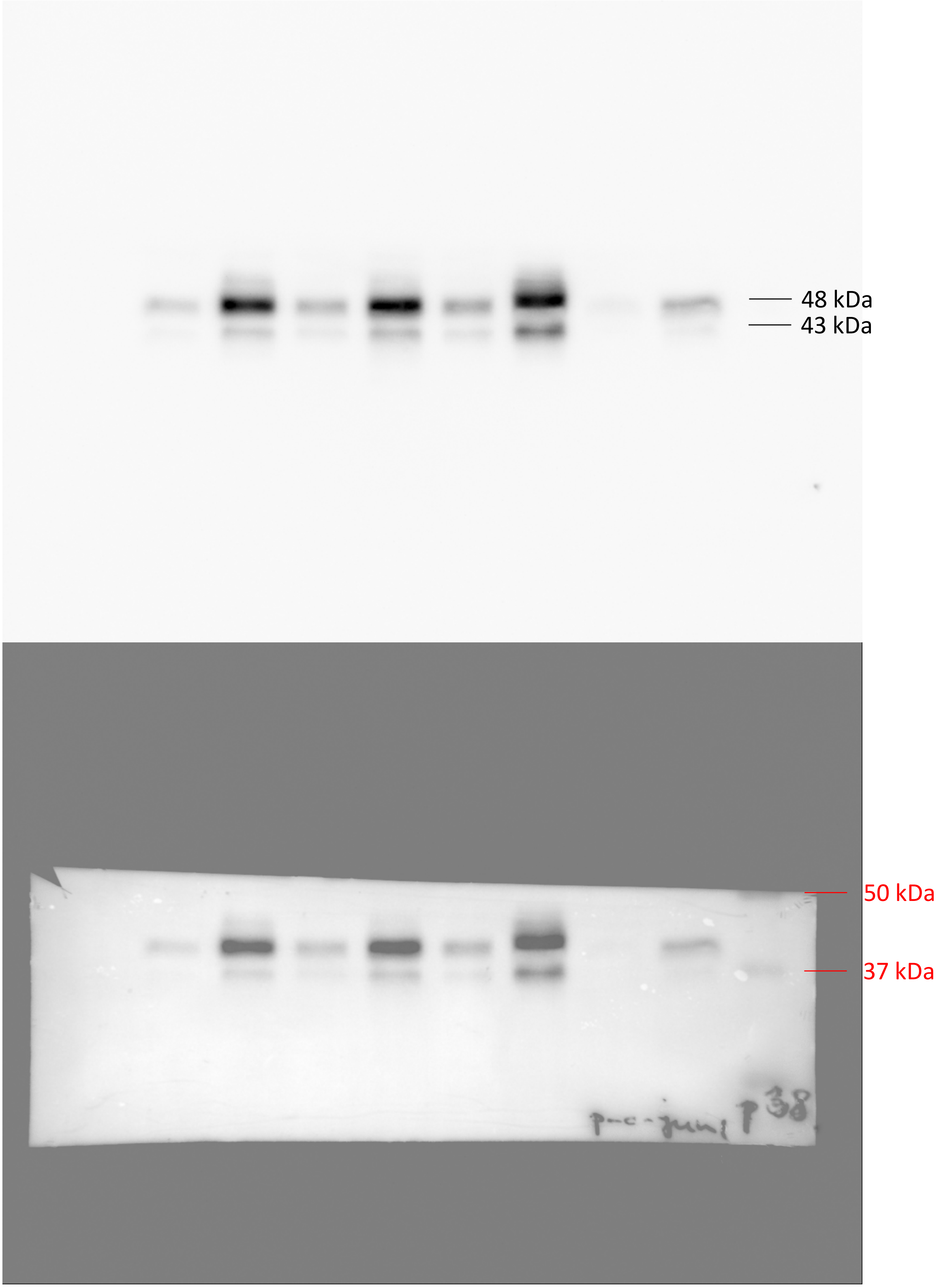

Fig. 4 Jun

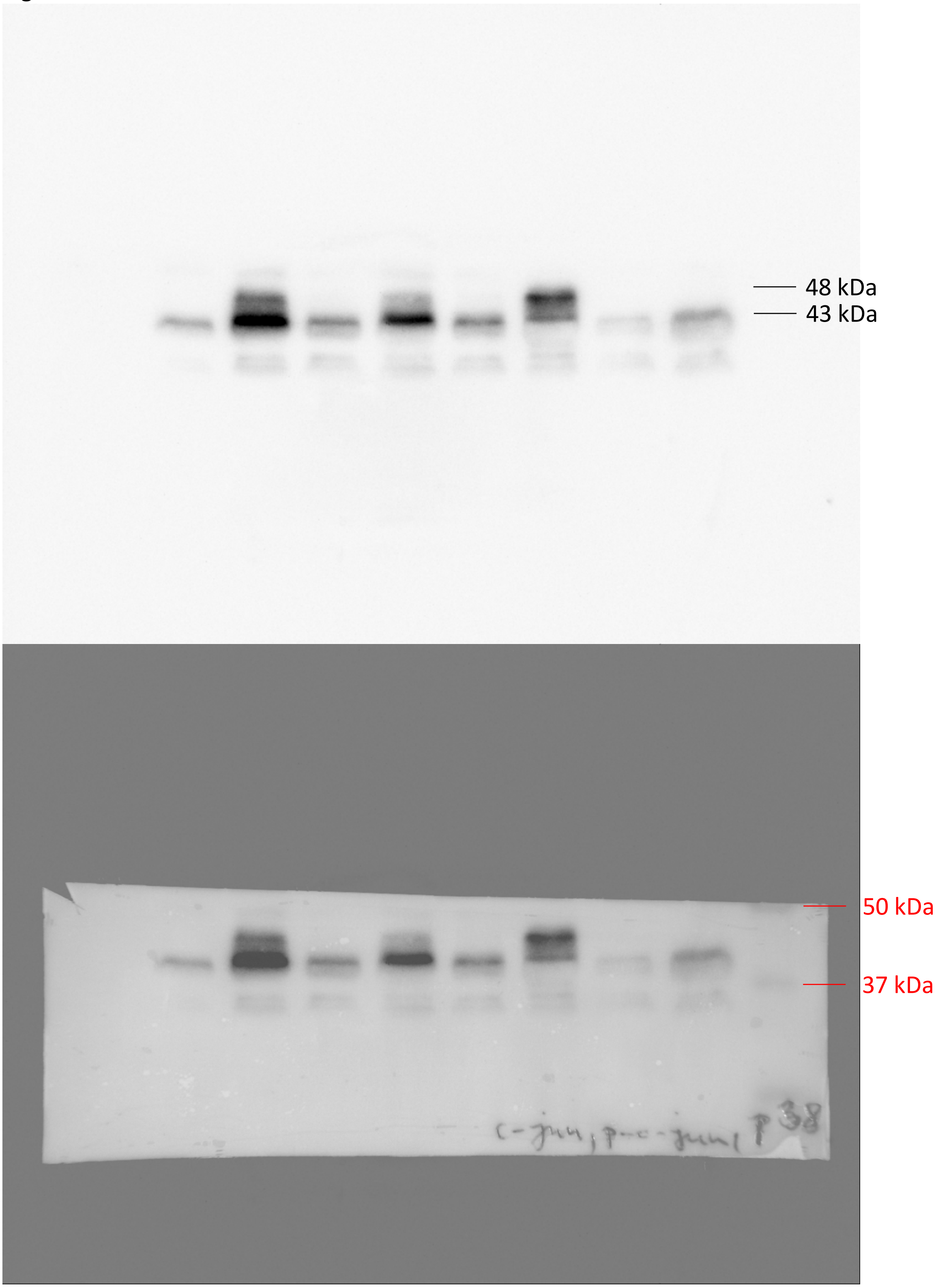

Fig. 4 GAPDH

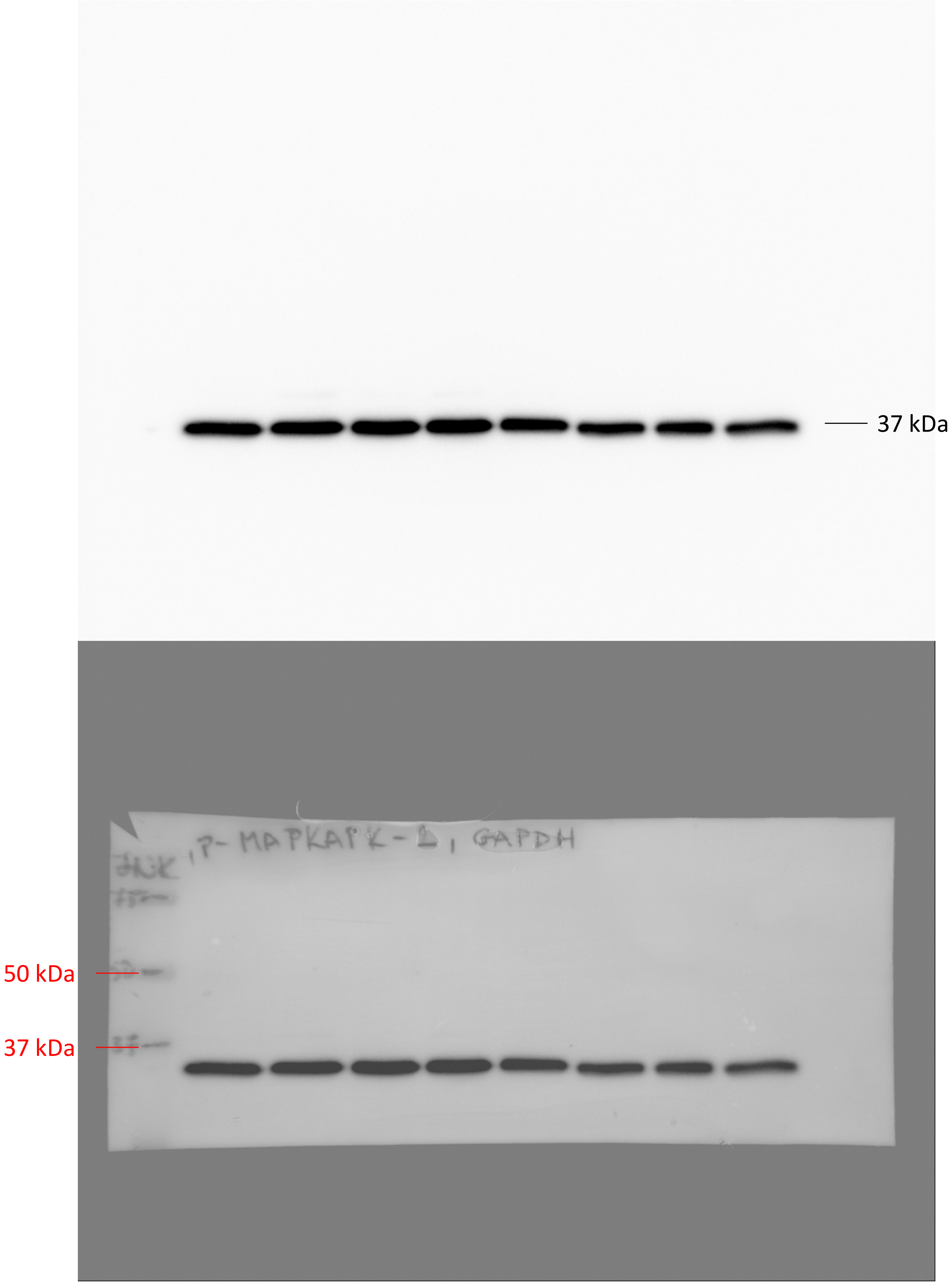

Fig. 5 p-Akt

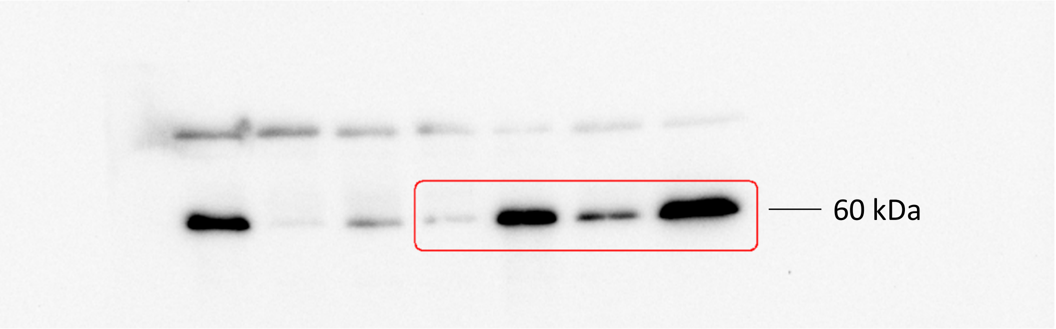

Fig. 5 Akt

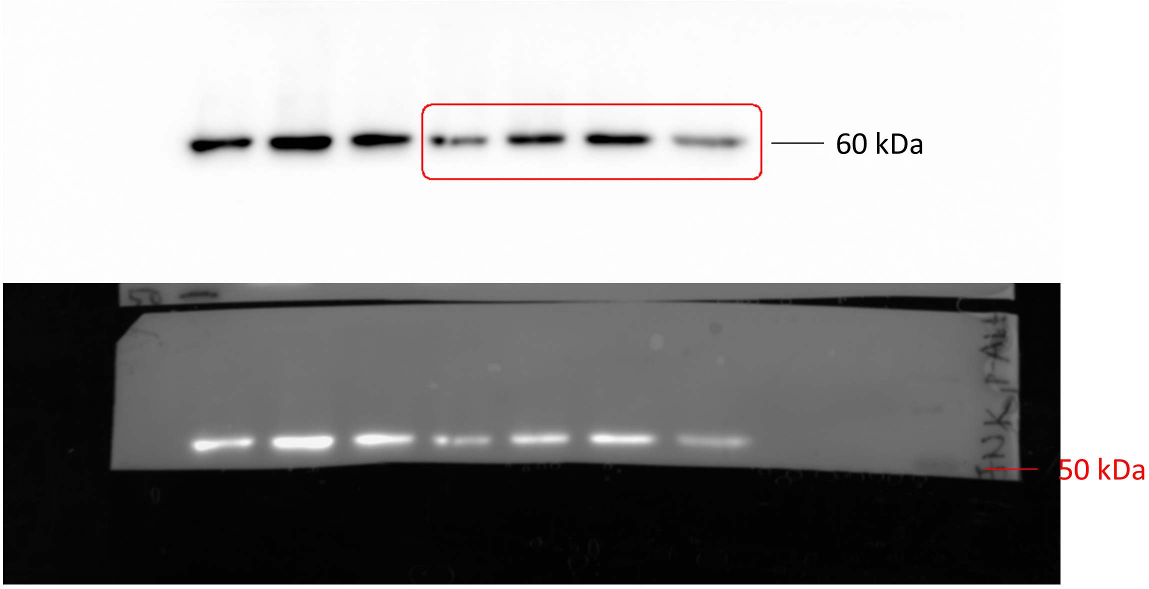

Fig. 5 p-p38

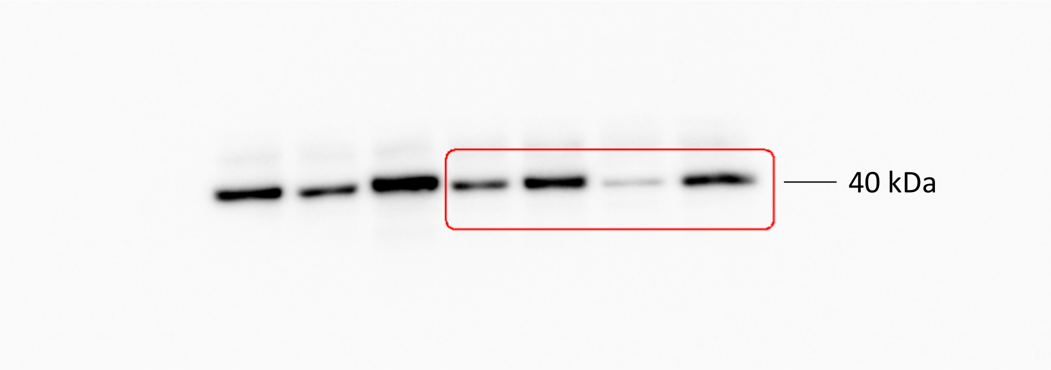

Fig. 5 p38

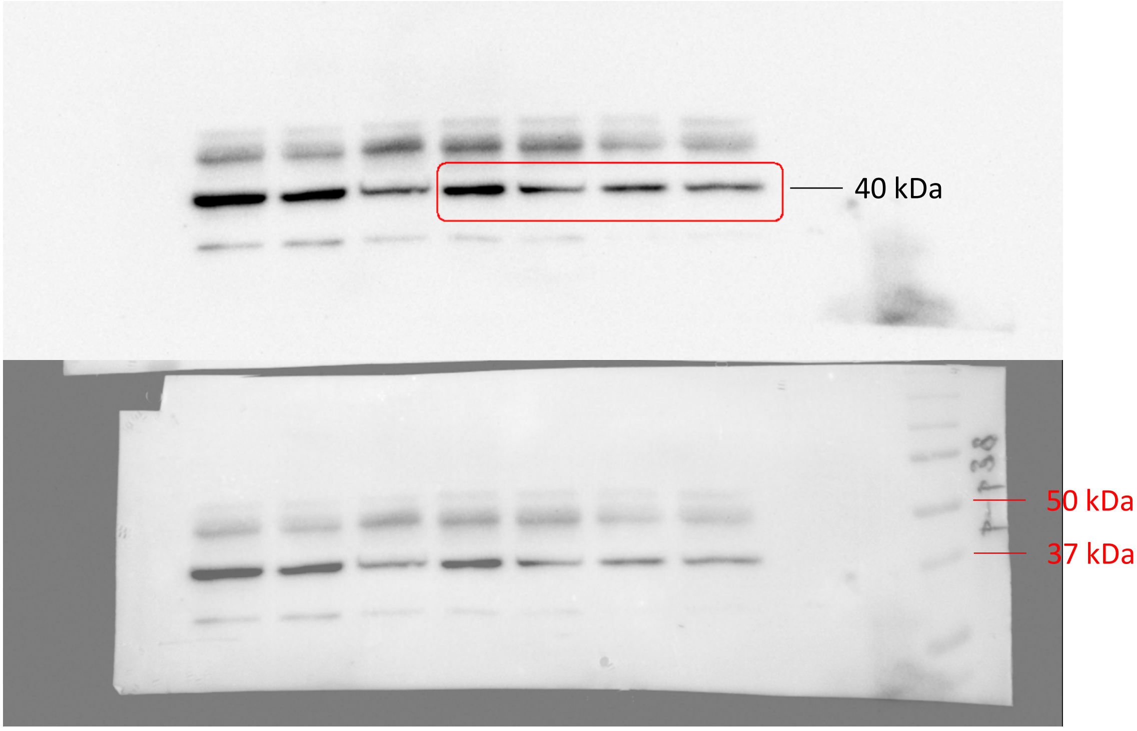

Fig. 5 p-JNK

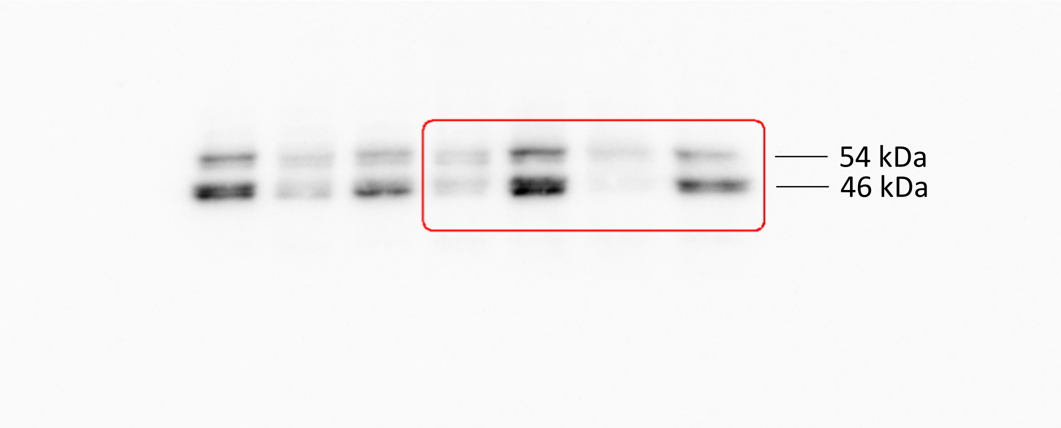

Fig. 5 JNK

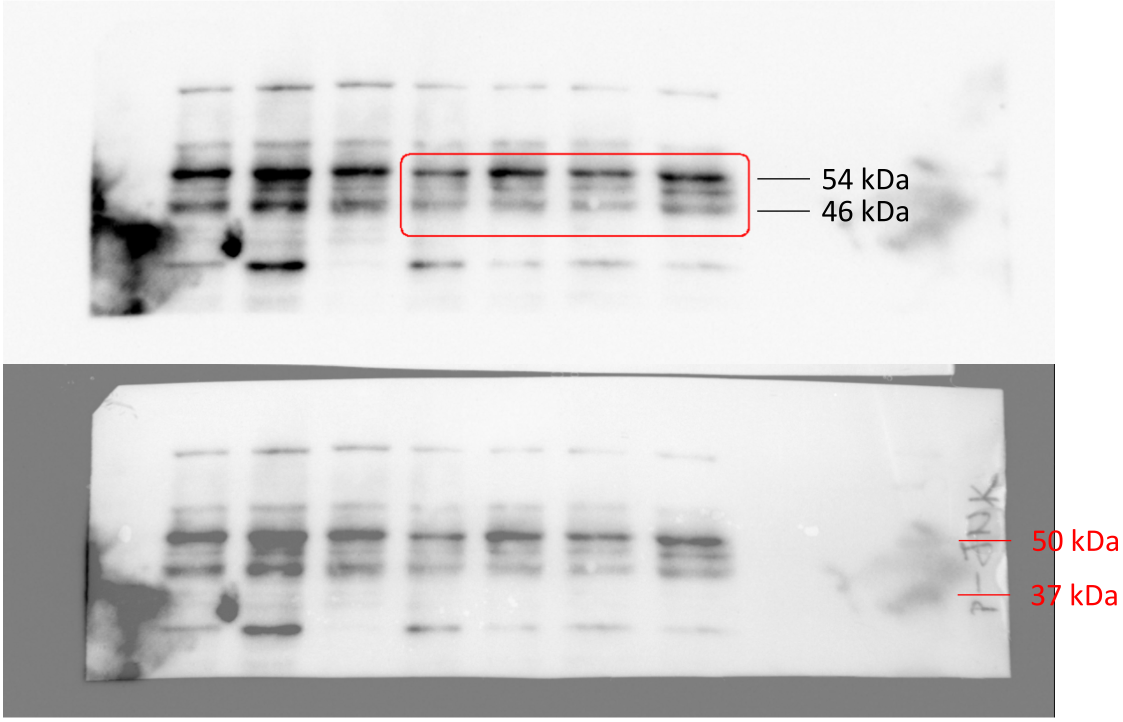

Fig. 5 p-Jun

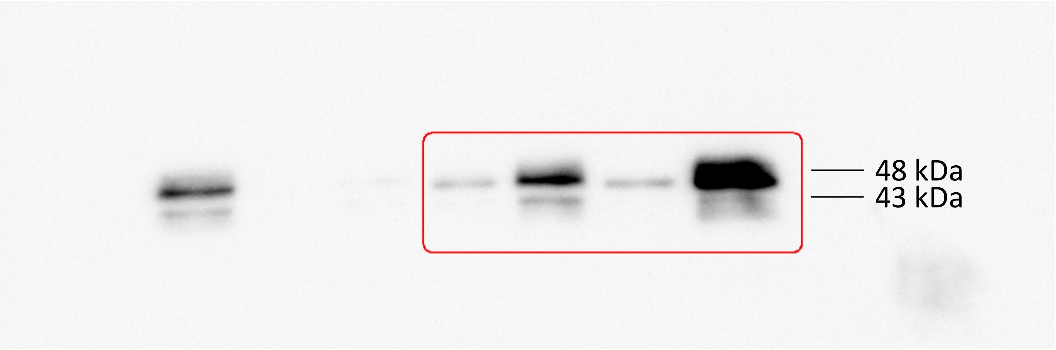

Fig. 5 Jun

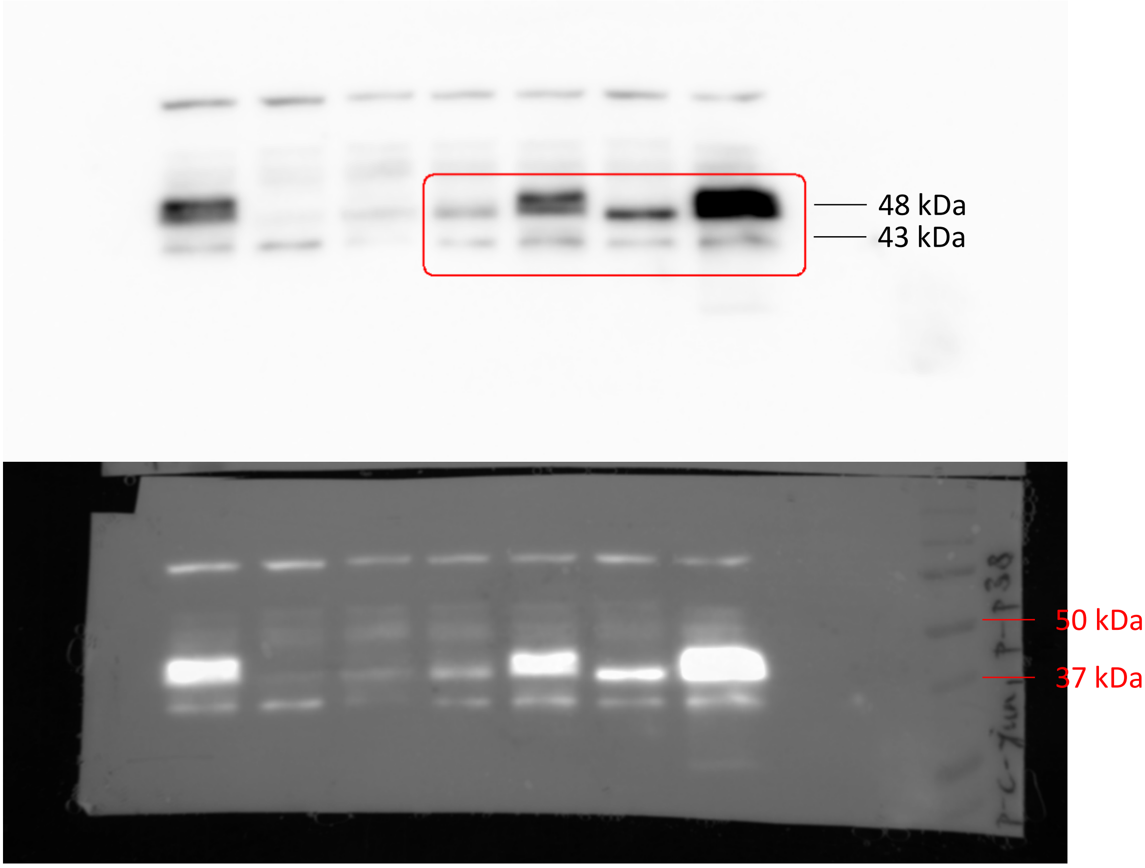

Fig. 5 GAPDH

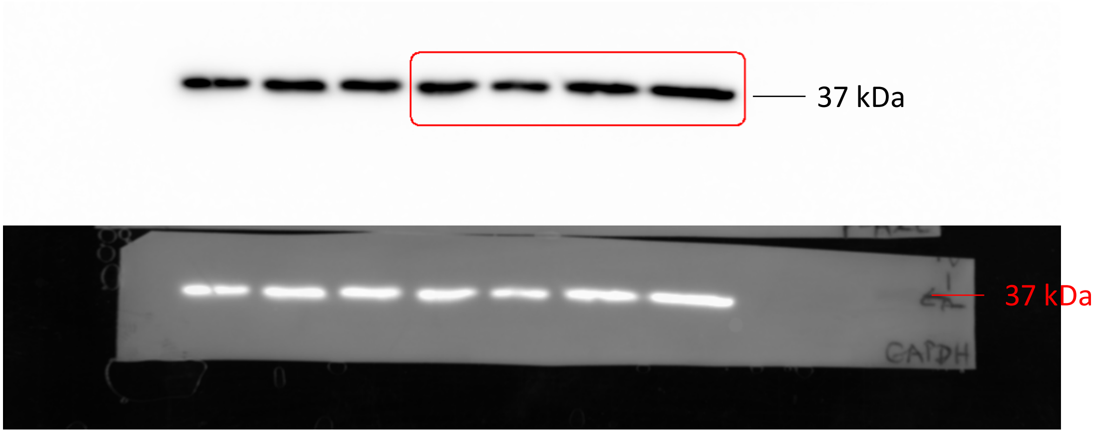

Suppl. Fig. 2 p-Akt

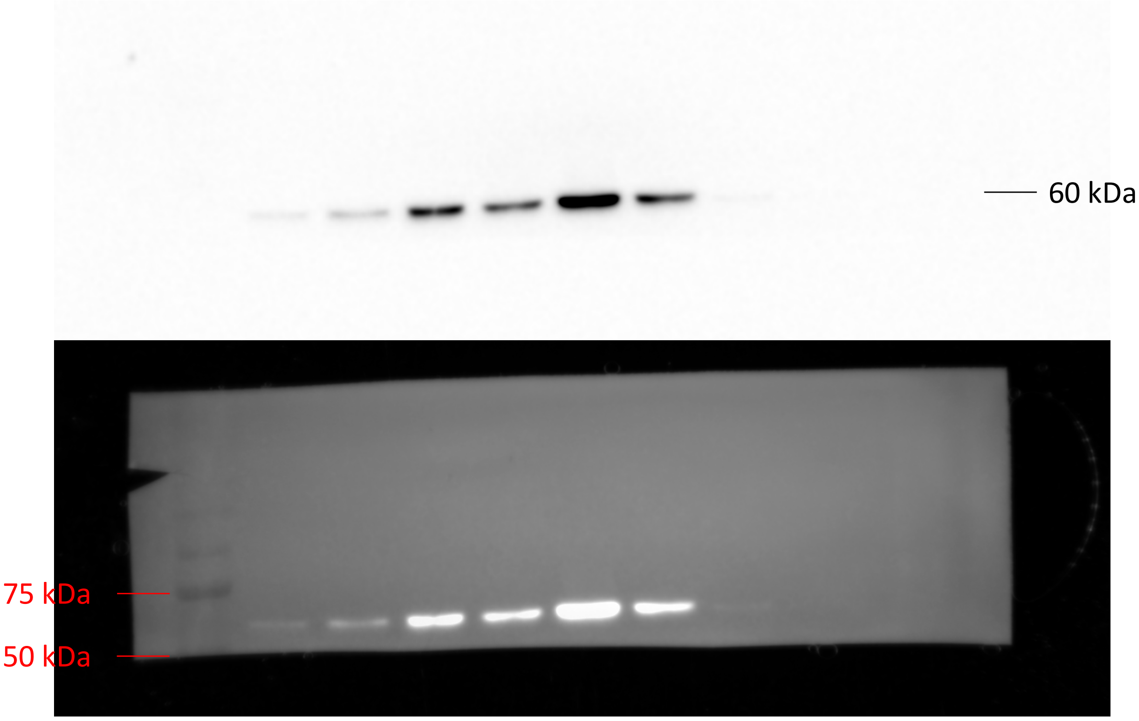

Suppl. Fig 2 Akt

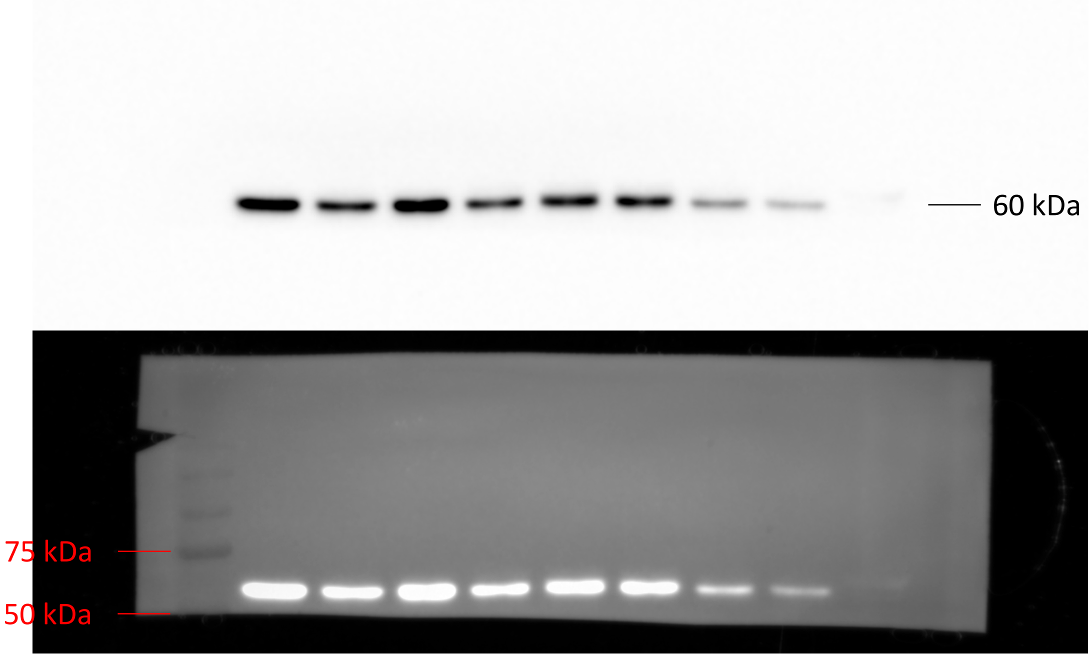

Suppl. Fig. 2 p-p38

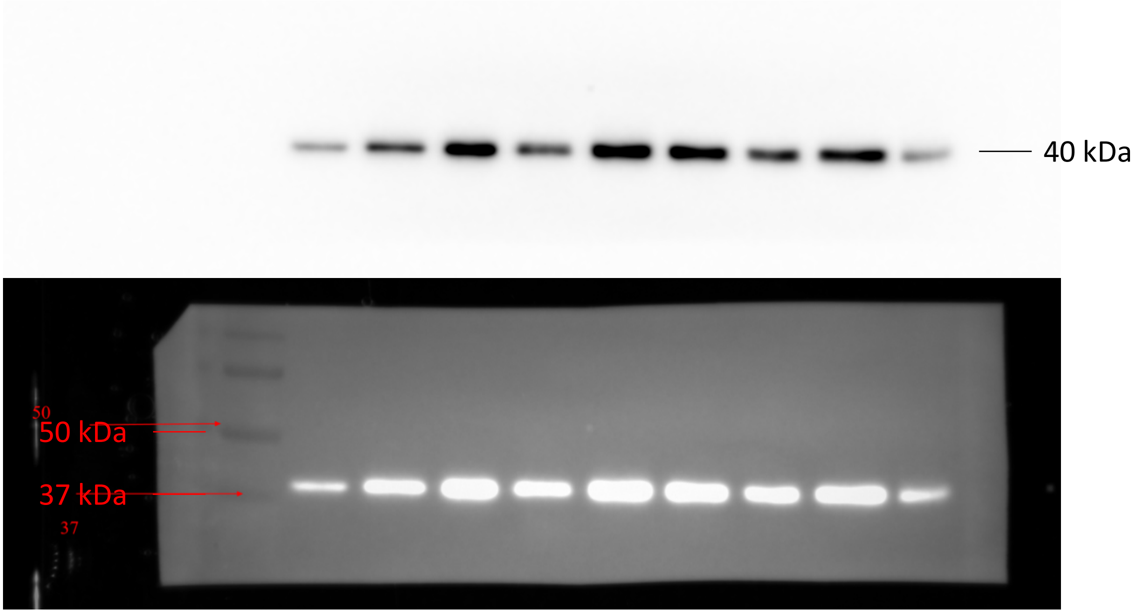

Suppl. Fig. 2 p38

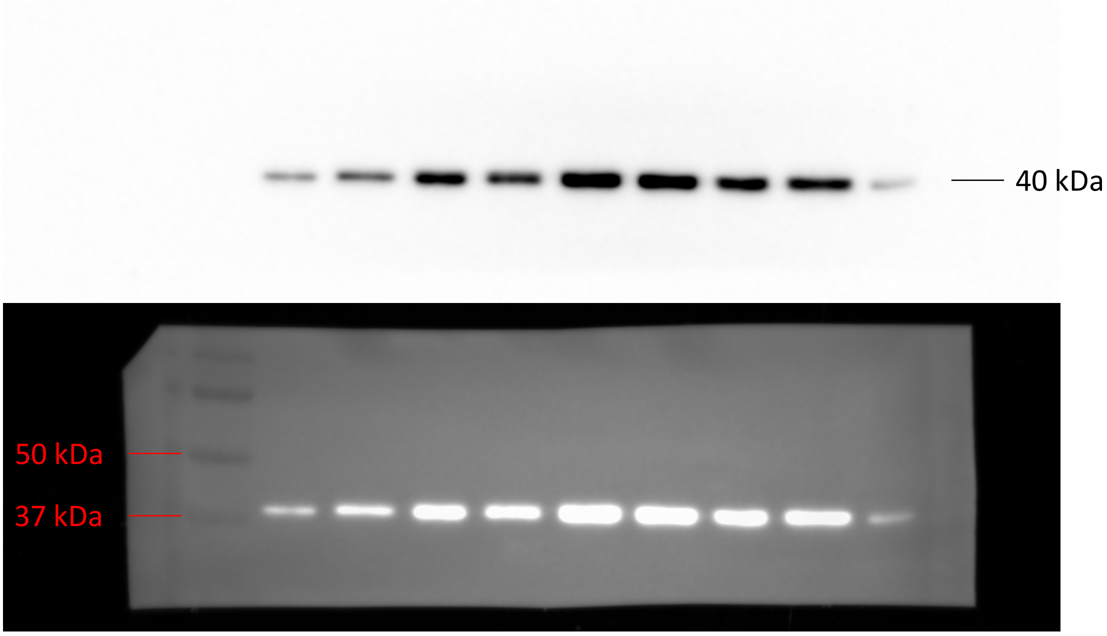

Suppl. Fig. 2 p-JNK

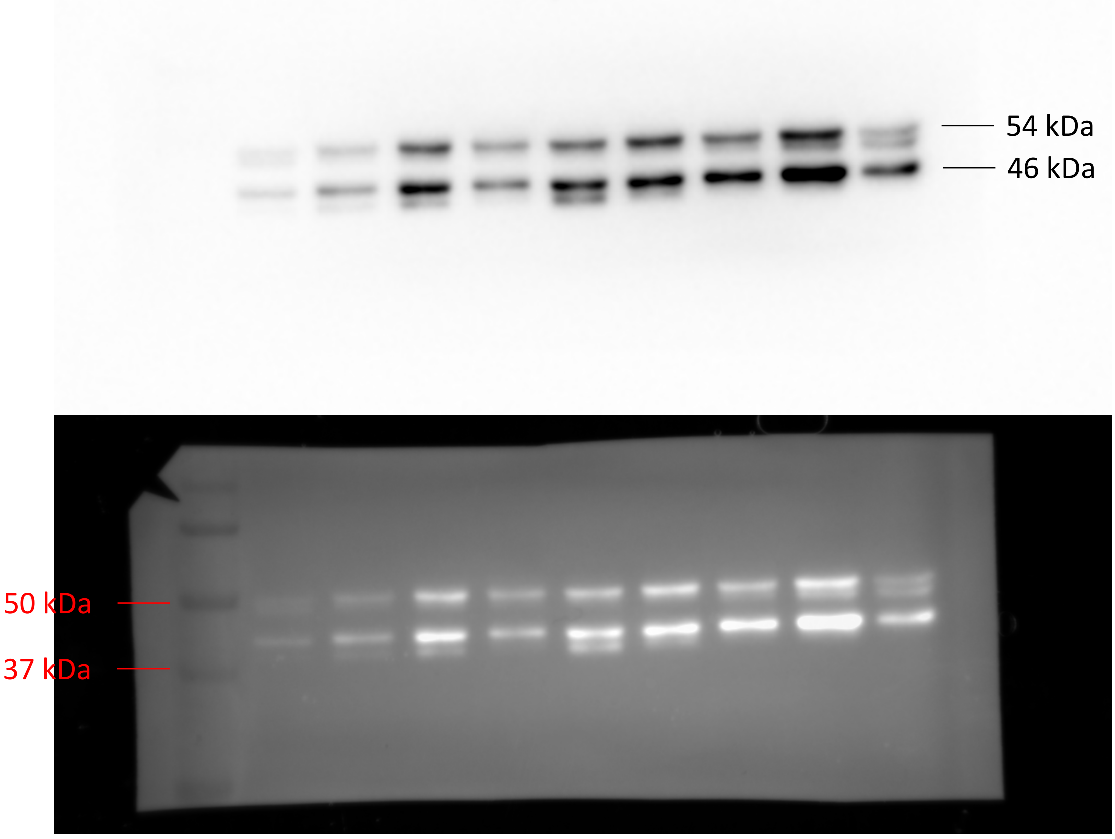

Suppl. Fig. 2 JNK

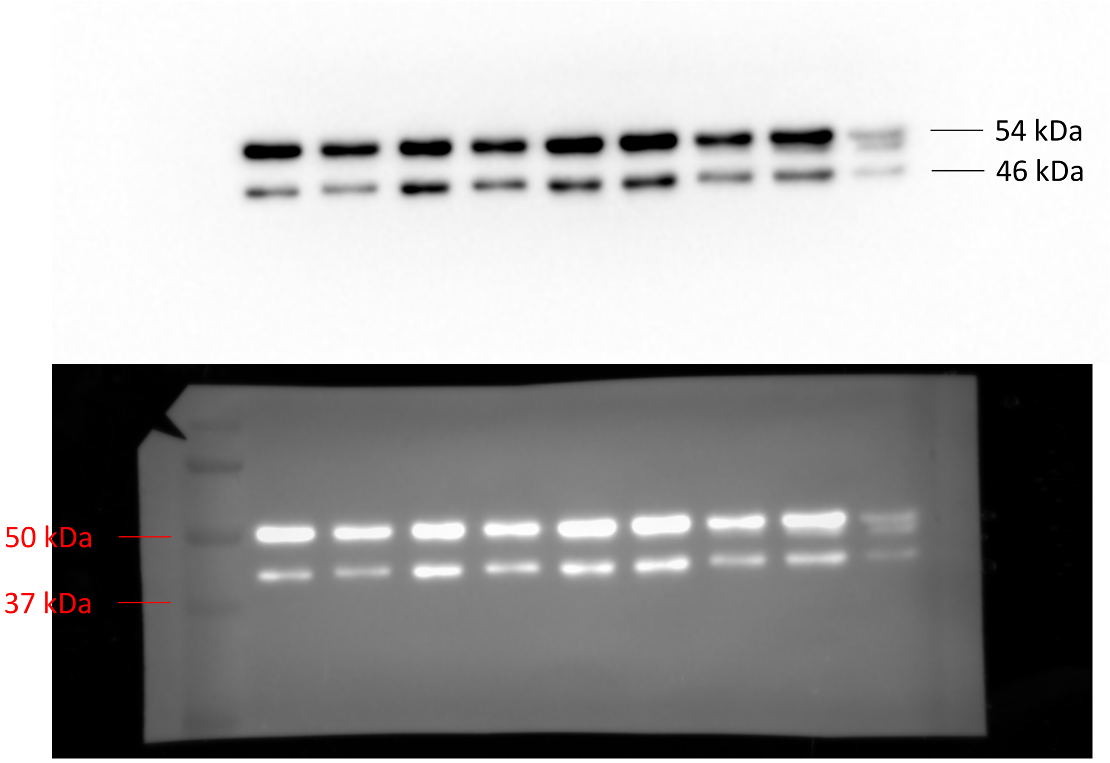

Suppl. Fig. 2 p-Jun

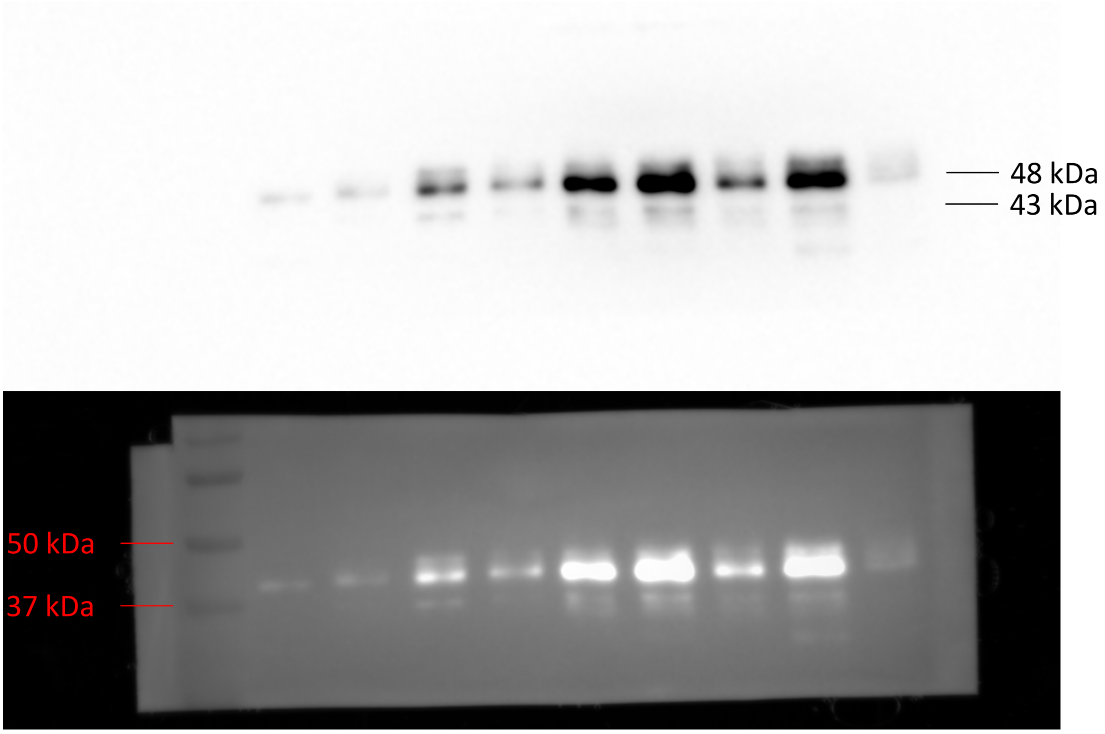

Suppl. Fig. 2 Jun

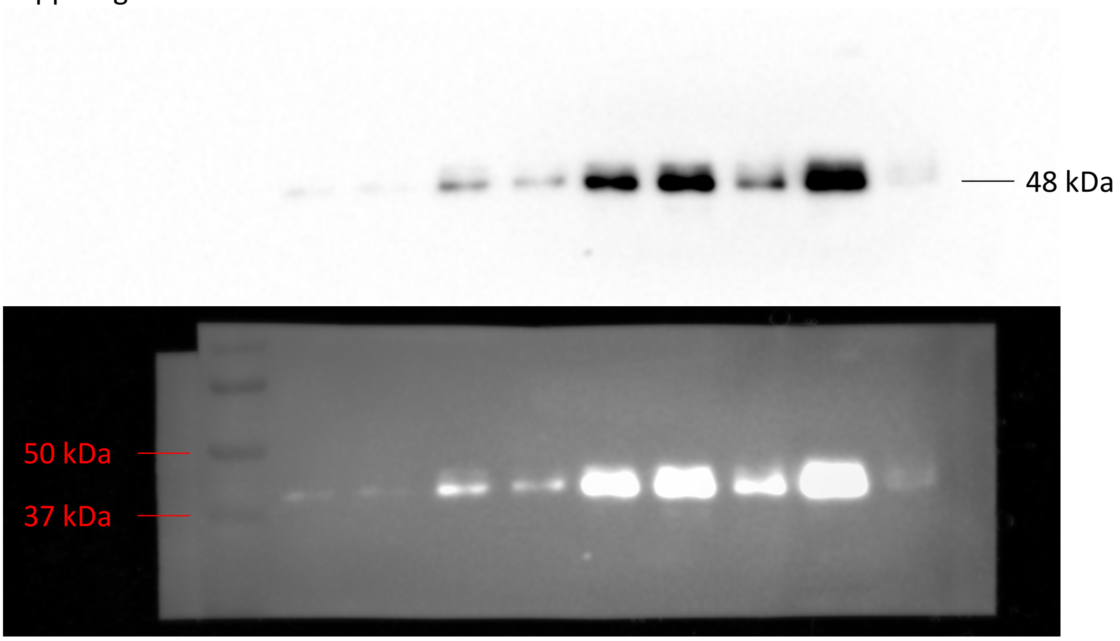

Suppl. Fig. 2 GAPDH

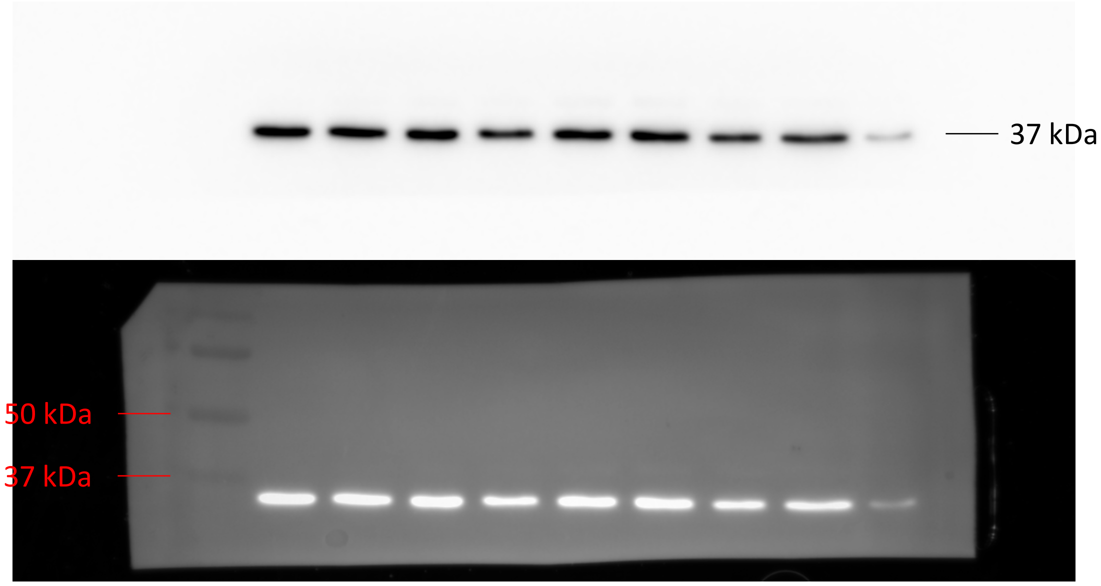

Suppl. Fig. Casp3

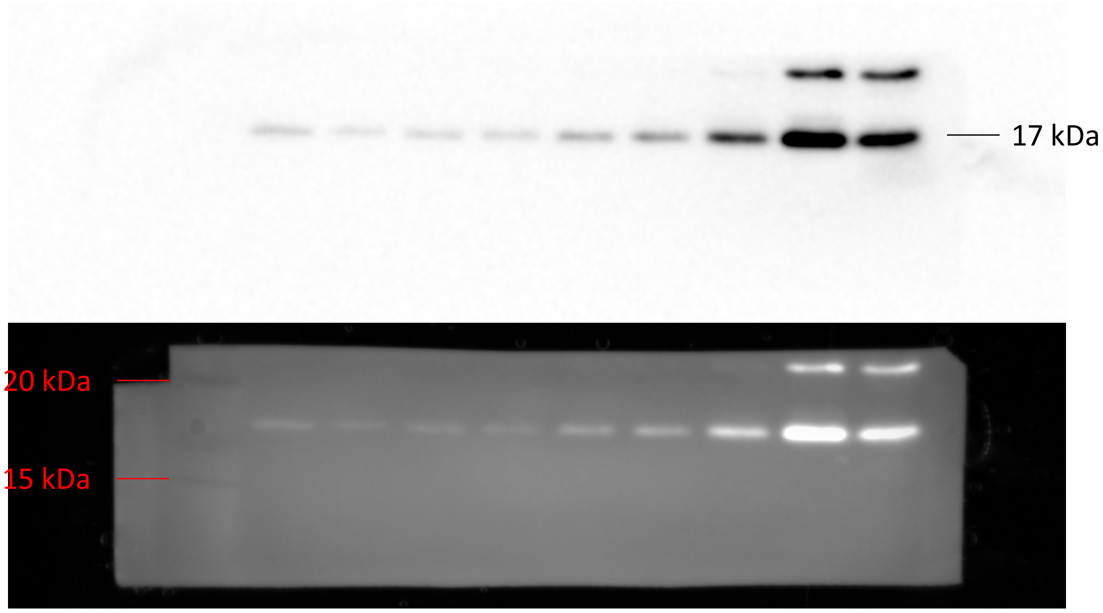

Suppl. Fig. 2 GAPDH for Casp3

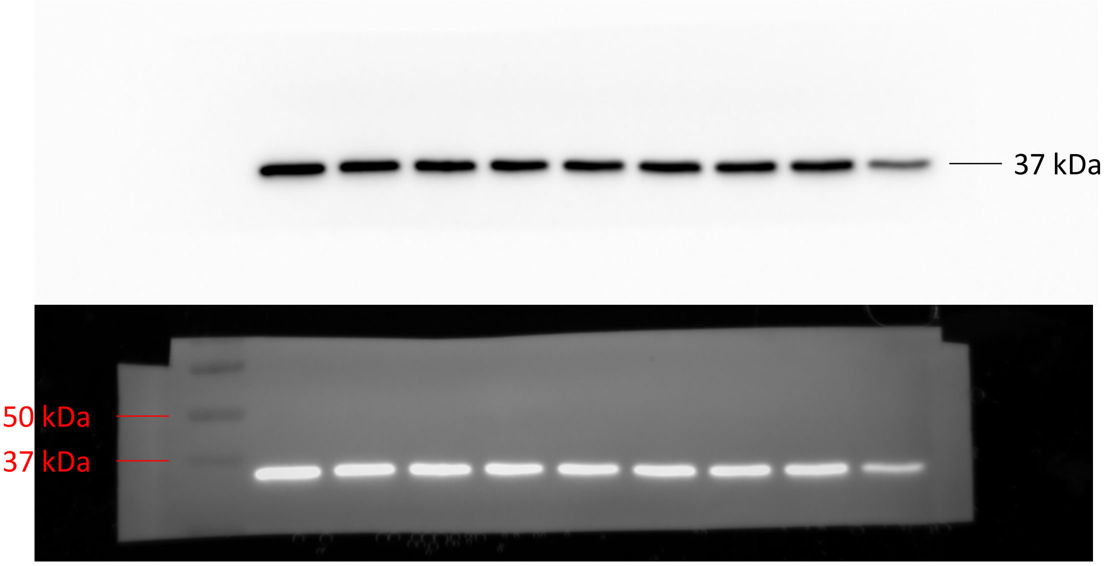

Supplement: Supplementary file 1 — Supplementary Information 1. [file 41598_2022_9763_MOESM1_ESM.pdf]
