# Supplementary figures and images for: Prolonged treatment with the proteasome inhibitor MG-132 induces apoptosis in PC12 rat pheochromocytoma cells
Source: Sci Rep. 2022 Apr 6;12:5808. doi: 10.1038/s41598-022-09763-z (PMC8987075; doi:10.1038/s41598-022-09763-z)

Suppl. Fig. 1

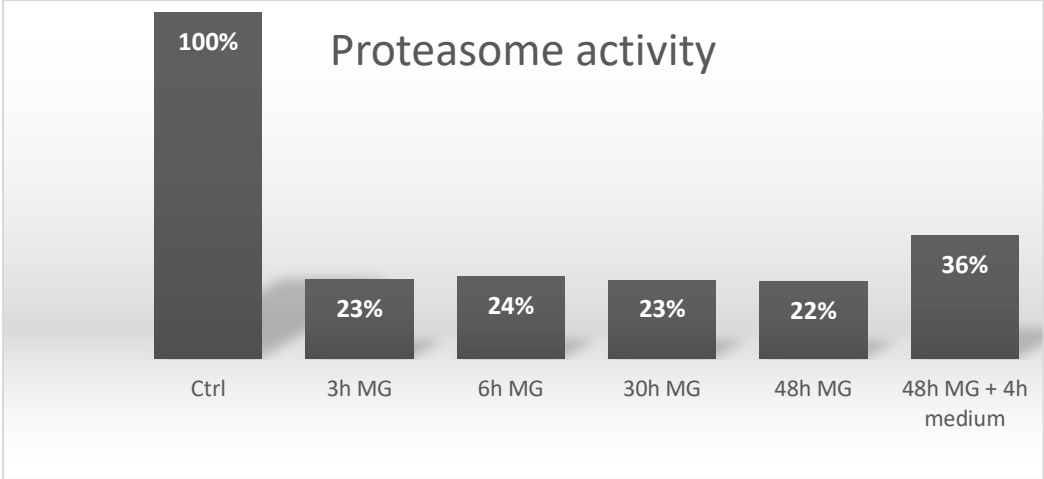

Suppl. Fig. 2

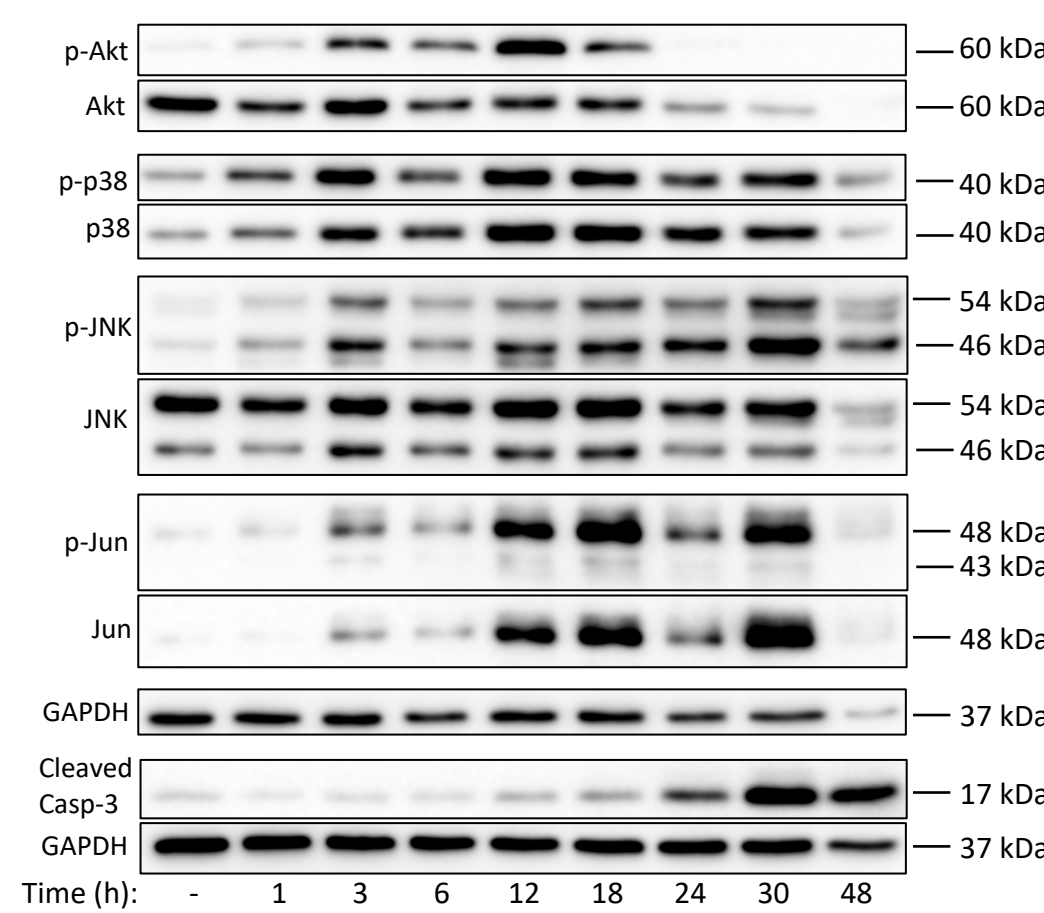

**Suppl. Fig. 3**

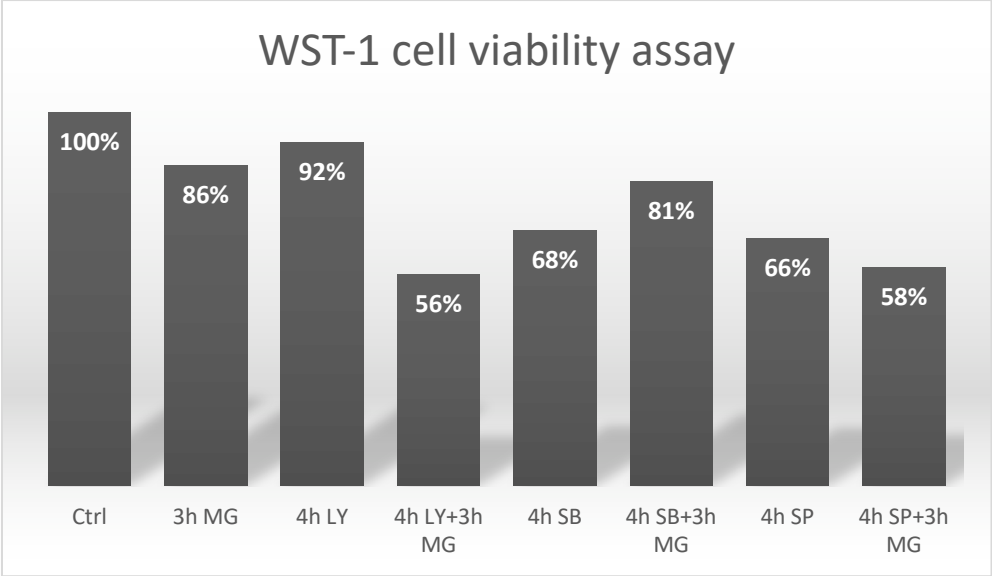

Supplement: Supplementary file 2 — Supplementary Information 2. [file 41598_2022_9763_MOESM2_ESM.pdf]
